# Integrating single-cell and bulk transcriptomes to reveal prognostic and immunological features of ecDNA-related genes in osteosarcoma

**DOI:** 10.1007/s00262-026-04383-2

**Published:** 2026-04-28

**Authors:** Jinyan Feng, Jianchao Liu, Houzhi Yang, Yiqin Li, Yao Xu, Xiuxin Han, Chao Zhang, Guowen Wang

**Affiliations:** 1 Department of Bone and Soft Tissue Tumors, Tianjin Medical University Cancer Institute and Hospital, Tianjin, 300060 People’s Republic of China; 2 State Key Laboratory of Druggability Evaluation and Systematic Translational Medicine, National Clinical Research Center for Cancer, Tianjin, 300060 People’s Republic of China; 3 Tianjin Key Laboratory of Cancer Prevention and Therapy, Tianjin’s Clinical Research Center for Cancer, Tianjin, 300060 People’s Republic of China

**Keywords:** ecDNA-related genes, Single-cell RNA sequencing, Machine learning, Osteosarcoma, Tumor immune microenvironment

## Abstract

**Background:**

The role of extrachromosomal DNA (ecDNA)-related genes in osteosarcoma remains largely unexplored. The aim of this study is to investigate the association between ecDNA-related genes and prognosis and tumor microenvironment (TME) in osteosarcoma.

**Methods:**

Differential gene expression analysis of GEO datasets was conducted to identify ecDNA-related genes in osteosarcoma. Based on bulk RNA-seq data, a novel ecDNA-related Gene Prognostic Score Model (EGPSM) was developed using an integrated framework of 101 machine learning algorithms, which was validated in training, testing, and external cohorts. The associations between risk scores, prognosis, and TME characteristics were comprehensively evaluated. Single-cell RNA sequencing (scRNA-seq) data were further analyzed to elucidate the relationship between EGPSM, pro-tumor behaviors, and immune modulation in osteosarcoma, as well as to identify key prognostic genes involved in tumor progression. Lastly, we conducted in vitro and in vivo assays to characterize the biological roles of MTDH and to elucidate its regulatory effects on CD8⁺ T cell function.

**Results:**

A robust EGPSM was constructed, demonstrating superior predictive accuracy with a maximum C-index of 0.803. High-risk patients exhibited poorer survival, higher metastatic potential, and an "immune-cold" TME characterized by diminished CD8⁺ T/NK cell infiltration and impaired effector functions. Single-cell analysis confirmed the enrichment of malignant cells and depletion of T/NK populations with lower effector scores in the high-risk group. MTDH was identified as a key driver; functional assays showed it promotes proliferation and invasion while inhibiting apoptosis. Notably, MTDH knockdown potentiated CD8⁺ T-cell cytotoxicity by increasing the levels of granzyme B, IFN-*γ*, and perforin.

**Conclusion:**

The newly developed EGPSM represents an effective tool for prognostic assessment and therapeutic stratification in osteosarcoma. MTDH may serve as a promising prognostic biomarker and therapeutic target.

**Supplementary Information:**

The online version contains supplementary material available at 10.1007/s00262-026-04383-2.

## Introduction

Extrachromosomal DNA (ecDNA), a distinct type of acentric, circular DNA found exclusively in tumors, was first observed in 1965 [1]. Double-strand DNA breaks, asymmetric chromosome segregation, micronucleus formation, and the breakage-fusion-bridge (BFB) cycle are all mechanisms for the formation of these DNA fragments[2–4]. EcDNA plays a crucial role as an important mechanism for gene amplification [5]. EcDNA contains oncogenes, gene enhancers, and immunoregulatory genes, and its length can reach several megabase pairs (Mbp) [6]. Due to the absence of a centromere, ecDNA undergoes unequal segregation during mitosis, a property which facilitates its rapid accumulation within cancer cells. [7]. Tumors utilize ecDNA to achieve a higher oncogene copy number than is possible on chromosomal DNA, driving extreme oncogene expression [8]. Alterations in oncogene dosage drive tumor progression and heterogeneity [9, 10]. For decades, ecDNA hidden in large-scale genomic data was largely undetected. With the benefit of the development of new technologies, our understanding of ecDNA has advanced significantly in both breadth and depth over the past decade [11, 12]. EcDNA has been detected in most human cancer types. Recent research has uncovered ecDNA’s significant roles in tumor development. These include promoting oncogene expression, enhancing intratumoral genetic heterogeneity, facilitating tumor adaptation, and correlating with poorer patient prognosis [13]. Specifically, the presence of ecDNA-related genes is linked to poor prognosis not only through ultra-high focal amplification and genomic instability, but also via the modulation of the tumor microenvironment. Studies have reported that ecDNA can impede T-cell infiltration, thereby accelerating tumor growth[7, 14]. Recent pioneering pan-cancer studies using TCGA data have demonstrated that ecDNA-containing tumors exhibit an ‘immune-cold’ phenotype, characterized by reduced immune infiltration and diminished antigen presentation[6]. Notably, the positive selection of immunomodulatory genes on ecDNA facilitates immune evasion, further distinguishing its biological impact from general copy-number variations. These findings indicate the significant potential of ecDNA as a cancer diagnostic biomarker and therapeutic target.

With an incidence of 3–4.5 cases per million population, osteosarcoma mainly affects adolescents and young adults and is the most common primary malignant bone tumor[15, 16]. This kind of tumor has strong invasion, early metastasis, high disability rate and poor prognosis. In recent decades, the treatment of osteosarcoma has entered a plateau, and traditional treatment methods are difficult to break through [17]. Although the 5-year survival rate for patients with localized osteosarcoma exceeds 78%, the 5-year survival rate for patients with metastatic or recurrent osteosarcoma drops to 25% [18]. However, combination immunotherapy, including immune checkpoint blockade inhibitors, has limited therapeutic effect in patients with osteosarcoma [19, 20]. Latest research in tumor immunology reveals that osteosarcoma, while long considered an “immune-cold” tumor, harbors a suppressive microenvironment that can be reprogrammed, and its immunogenicity still has the potential to be activated and enhanced [21, 22]. Structural variations (SVs) and copy number aberrations (CNAs) are the main features of osteosarcoma, which reflect its extensive genomic instability and chromosomal abnormalities [15, 23, 24]. Oncogene CNA plays a crucial role in the development of tumor [25]. Such amplifications typically present as focal, high-level gains and can be driven by ecDNA circular structures [9]. Current research on ecDNA in osteosarcoma is limited; however, due to its critical role in tumor progression and genomic instability, it is essential to explore the contribution of ecDNA to osteosarcoma.

In this study, a novel ecDNA-related Gene Prognostic Score Model (EGPSM) was developed using integrated machine learning and validated across multiple cohorts, demonstrating strong predictive power for osteosarcoma prognosis. High-risk patients showed poorer survival, higher metastatic potential, and an “immune-cold” tumor microenvironment characterized by reduced T/NK cell infiltration and increased T-cell dysfunction. Single-cell analysis identified MTDH as highly expressed in osteosarcoma cells, promoting proliferation, migration, and invasion while inhibiting apoptosis. Moreover, MTDH knockdown enhanced CD8⁺ T-cell cytotoxicity, restoring anti-tumor immunity and suppressing tumor growth. Collectively, these findings contribute to a deeper understanding of the mechanism of ecDNA in the occurrence and development of osteosarcoma, and indicate potential prognostic and therapeutic targets for the treatment of osteosarcoma.

## Materials and methods

### Datasets preparation

We conducted a comprehensive and integrated analysis using osteosarcoma single-cell RNA sequencing (scRNA-seq) and bulk RNA sequencing (bulk RNA-seq) datasets. The scRNA-seq data were acquired from the Gene Expression Omnibus (GEO) database (GSE162454; https://www.ncbi.nlm.nih.gov/geo/) and generated using the 10× Genomics platform. This dataset comprises transcriptomic profiles from six primary, treatment-naive osteosarcoma specimens [26]. For bulk RNA-seq analysis, publicly available cohorts with both gene expression profiles and complete clinical follow-up information were retrieved from the GEO and TARGET (Therapeutically Applicable Research to Generate Effective Treatments) repositories (https://www.cancer.gov/ccg/research/genome-sequencing/target). These included GSE21257 (*n* = 53), GSE39055 (*n* = 37), GSE33382 (*n* = 82), and TARGET-OS (*n* = 84). We performed standardized processing on the datasets used in this study. All raw data were converted into a uniform format (e.g., TPM), followed by a log_2_(TPM+1) transformation. Subsequently, batch effect correction was implemented using the “sva” R package and the removeBatchEffect function from the “limma” R package. This refined expression matrix served as a robust foundation for the subsequent construction of the 101 machine-learning combinations.

### Differential and functional analysis

We used “limma” R package to explore differentially expressed genes (DEGs) between osteosarcoma and normal tissues in R (R 4.4.0) Software. DEGs were identified using a cutoff of |log2 (fold change) |> 2 and *p* < 0.05. Gene Ontology (GO) and Kyoto Encyclopedia of Genes and Genomes (KEGG) pathway enrichment analyses were performed on the differentially expressed genes (DEGs). Several R packages, including “clusterProfiler,” “org.Hs.eg.db” and “enrichment plot” were used for functional analysis. A *p value* of < 0.05 was significantly enriched.

### Construction of EGPSM

We developed a prognostic signature following an established framework integrating 101 machine learning (ML) combinations derived from 10 classic algorithms[27–29]. These algorithms included least absolute shrinkage and selection operator (LASSO), gradient boosting machine (GBM), random survival forest (RSF), cox partial least squares regression (plsRcox), stepwise cox regression (StepCox), supervised principal components (SuperPC), Ridge Regression, survival support vector machine (Survival-SVM), CoxBoost, and Elastic Network (Enet). GSE21257 was utilized as the training set, while GSE33382 and GSE39055 served as the testing set and the external validation set, respectively. To eliminate potential human bias and ensure objectivity in model construction, we implemented an automated hyperparameter tuning pipeline within a nested tenfold cross-validation framework. Specifically, for each of the 101 ML combinations, optimal parameters were automatically determined through a systematic grid search within a predefined parameter space. For instance, the penalty parameter *λ* in LASSO-related models was selected based on an automated cv. glmnet procedure to minimize cross-validation error, while forest-related parameters in RSF and GBM (e.g., mtry and nodesize) were optimized via an automated search aimed at maximizing the Harrell’s concordance index (C-index). This non-manual, data-driven approach ensures that our EGPSM maintains robustness and reproducibility across independent cohorts.

### Tumor immune infiltration analysis

The CIBERSORT gene expression deconvolution tool was employed to quantify the relative abundance of immune cell subsets. This information was then used to assess the relationship between the expression of the model genes and the resulting immune cell infiltration status.[30]. To assess immune activity in two subgroups of osteosarcoma samples and reveal differences in the tumor microenvironment (TME) between different risk groups, single-sample Gene Set Enrichment Analysis (ssGSEA) was performed. Subsequently, ESTIMATE analysis was utilized to calculate immune-related scores. Finally, a set of genes including immune checkpoints, chemokines, interleukins, interferons, receptors, and other cytokines, which were compiled from prior published studies, were utilized to assess the differential expression levels between the various risk groups [31].

### Prediction of drug sensitivity and immunotherapy

The TIDE online tool(http://tide.dfci.harvard.edu/) was employed to predict the potential clinical response of osteosarcoma patients to immunotherapy. A higher TIDE score indicates an increased likelihood of immune evasion, suggesting a lower probability of achieving a positive response to immune checkpoint inhibitors (ICIs). To investigate the relationship between different risk characteristics and drug sensitivity in osteosarcoma, we utilized the oncoPredict R package (https://github.com/HuangLabUMN/oncoPredict). This R package predicts the sensitivity of cancer patients to various drugs by comparing predicted IC50 values derived from the Genomics of Drug Sensitivity in Cancer (GDSC) database (version 2.0). Wilcoxon rank-sum tests were used to assess significant differences in the IC50 predictive values of each drug between the different groups.

### scRNA-seq data processing

Seurat (v5.0.0) R software package was used to systematically process osteosarcoma scRNA-seq data. We performed strict quality control by first excluding genes with fewer than 200 features detected in fewer than three cells. We further filtered cells with less than 300 or more than 5000 genes detected, and cells with more than 10% mitochondrial gene content. In addition, potential double cells were identified and excluded using the “DoubletFinder” R package. Batch effects were addressed using the “Harmony” (v1.2.3) R package. Subsequently, the UMAP dimensionality reduction method was used for visualization. Cell annotation was performed by means of established cell marker genes in previous articles[26, 32, 33]. InferCNV (v1.16.1) was applied to identify malignant cells by estimating chromosomal copy number variations. Specifically, within the InferCNV analysis, T/NK cells were selected as the reference group (baseline) to distinguish malignant osteosarcoma cells from non-malignant populations. In addition, the EGPSM score of each cell was calculated using the “AddModuleScore” function. We then divided the cells into high EGPSM score and low EGPSM score groups based on the threshold of the AddModuleScore method. The “Monocle 2.0 (v2.34.0)” R package was used to analyze the pseudo-time trajectory of osteosarcoma cell development. The DDRTree algorithm was employed to order cells along a pseudotime axis based on highly variable genes. Subsequently, we analyzed the dynamic expression of the EGPSM score along the inferred cellular trajectory. The “scMetabolism” R package was subsequently used to infer the metabolic pathway activity between the different groups and visualized using a heatmap. Cell-to-cell communication networks were inferred and visualized using the CellChat R package (v1.6.1). By constructing a ligand-receptor interaction network. The communication strength was then quantified by analyzing the overall number of interactions and the total signal strength. We performed virtual knockout analysis using the scTenifoldKnk R package (v1.0.1) to simulate the effects of gene deletion by perturbing gene–gene co-expression networks constructed from single-cell transcriptomic data. Briefly, a multi-layer gene co-expression network was first constructed from the control (wild type) expression matrix using principal component regression. A “knockout” network was then generated by setting all edge weights associated with MTDH to zero. By comparing the structural differences between the original and knockout networks using manifold alignment, we identified significantly affected genes, designated as virtual knockout differentially regulated genes. These DRGs were further utilized for functional enrichment analysis to infer the downstream biological consequences of MTDH deficiency.

### Cell culture and cell transfection

Both 143B and U2OS cells were obtained from the Chinese Academy of Sciences Cell Bank (Shanghai, China), and each cell line was grown in DMEM (Cellmax, Beijing, China) supplemented with 10% fetal bovine serum (FBS, Cellmax) and 1% penicillin/streptomycin. The used MTDH silencing RNA (si-MTDH) and the corresponding negative control siRNA were obtained from Sangon Biotechnology Co, ltd (Shanghai, China). All siRNAs were obtained from Sangon Biotech (Shanghai, China), and detailed sequence information is provided in Supplementary Table S2. Following the manufacturer’s instructions, 143B and U2OS cells were transfected with MTDH siRNA using PEI transfection reagent (40815ES03, YEASEN, China).

### Cell proliferation and colony formation assays

A total of 5 × 10^3^ 143B or U2OS cells were plated in 96-well plates. Cell proliferation was assessed using the CCK-8 assay (40203ES, Yeasen, China). After incubation for 1 h at 37° C, the absorbance of each well at 450 nm was detected. The cells were digested with trypsin and counted under a microscope and seeded into 6-well plates at 500 cells/well and subsequently cultured for 14 days. The cells were digested with trypsin and counted under a microscope and seeded into 6-well plates at 500 cells/well and subsequently cultured for 14 days. Colonies were fixed with absolute ethanol and finally stained with 0.1% crystal violet.

### Transwell migration and invasion assays

Cell migration and invasion were analyzed in 24 well plates using Transwell chambers with or without matrigel coating (12,100,089, LABSELECT, China). The cells were seeded in the upper chambers in serum-free medium without matrigel and in serum-free medium with matrigel, with the lower chamber medium containing 10% FBS. The migrated cells were stained, imaged, and counted after 24 h.

#### Cell apoptosis assay

Apoptosis was assessed using Annexin V-APC/PI double staining kit (40302ES50, YEASEC, China). Cells were digested with trypsin without EDTA and centrifuged at 300 × g for 5 min. Subsequently, the cells were washed twice with PBS and collected by centrifugation at 300 × g for 5 min. After the precipitate was retained, the cells were gently resuspended by adding binding buffer (500*μ*L). The cells were stained with 5*μ*L Annexin V-FITC and 10*μ*L PI and incubated in the dark for 20 min, and finally 400*μ*L binding buffer was added. Cell apoptosis was detected using BD FACSCanto II.

#### Wound healing assay

When cell confluence reached 90% to 100% in the 6-well plate, a 200 *μ*L pipette tip was used for vertical scratching at the bottom of the 6-well. Subsequently, the cells were washed twice with PBS to remove floating cells and then replaced with fresh serum-free medium and incubated at 37 °C in a volume fraction of CO2 of 5%. Pictures were taken under an inverted microscope at 0 and 12 h, and the scratch width was finally measured. The mean value of the distance between cells was calculated using Image J software.

#### T-cell-mediated tumor cell killing assays

Human PBMCS were purchased from Hycells Biotech (hPB050F, China), and T cells were purified from PBMCS with the use of CD3 magnetic beads. T cells were preactivated with CD3 antibody (100 ng/mL) (BioLegend, USA) and IL-2 (10 *μ*g/mL) (200-02-10UG, PeproTech, USA)[34]. Tumor cells were pre-treated in advance according to the corresponding grouping and subsequently co-cultured with activated T cells at a ratio of 1:3 at 37 °C for 3 days [35]. Viable tumor cells were washed with PBS and stained with 0.1% crystal violet solution. T cells from different purpose groups after 48 h of co-culture were collected into 1.5 ml EP tubes, centrifuged at 400 × g for 5 min to collect T cells, and the supernatant was discarded. T cells were resuspended in FACS buffer, aliquoted at 200 *μ*L per tube into individual staining groups, and stained with antibodies against CD8 (BioLegend, USA), GZMB (BioLegend, USA), IFN-*γ* (BioLegend, USA), and perforin (BioLegend, USA). Finally, T cells were resuspended in FACS buffer and subjected to and flow cytometry analysis. Information on antibodies used in this article is provided in Supplementary Table S3.

#### In vivo animal experiments

All the animal experiments were approved by Tianjin Medical University Animal Care and Use Committee. Four-week-old female BALB/c mice were purchased from GemPharmatech Co., Ltd. (Jiangsu, China) and acclimated for one week prior to the experiments. A total of ten BALB/c mice were randomly assigned into two groups (*n* = 5 per group): sh-NC and sh-MTDH. To establish the orthotopic osteosarcoma model, 5 × 105 K7M2/luc tumor cells (10 ul) were aspirated using a micro-syringe and transplanted into the right proximal tibial plateau of each mouse. After four weeks of observation, bioluminescence imaging (BLI) was performed using an in vivo imaging system for quantitative analysis of tumor progression. Mice were euthanized, and the tumor-bearing hind limbs were amputated to isolate tumor tissues. The tumor tissues were minced using surgical blades and then enzymatically dissociated using a Tumor Dissociation Kit (Miltenyi Biotec, Bergisch Gladbach, Germany). Subsequently, these segments were incubated in an enzymatic cocktail containing 2.35 mL of RPMI 1640, 100 uL of enzyme D, 10 uL of enzyme R, and 12.5 uL of enzyme A at 37 °C for 1 h. Following dissociation, the suspension was filtered through a 70-um strainer to obtain single cells. Cells were first stained with corresponding antibodies for surface markers, then fixed with 4% paraformaldehyde and permeabilized using the Cyto-Fast Fix/Perm Buffer Set (BioLegend). Intracellular staining was then performed using antibodies against cytokines, including perforin, IFN-γ, and GZMB. Flow cytometry was performed using an LSRFortessa SORP flow cytometer (BD Biosciences, Franklin Lakes, NJ, USA), and data were analyzed using FlowJo software. Detailed antibody information is provided in Supplementary Table S3.

#### Statistical analysis

All experiments were independently repeated at least three times. R software (v 4.2.0) was used for bioinformatics analysis. Unless otherwise stated, differences between two groups were assessed using the Wilcoxon rank-sum test, whereas differences between more than two groups (multiple groups) were analyzed using one-way ANOVA (parametric) or Kruskal–Walli’s rank-sum test (nonparametric). *P* < 0.05 was considered statistically significant.

## Results

### Construction and validation of EGPSM

**Fig. 1:**
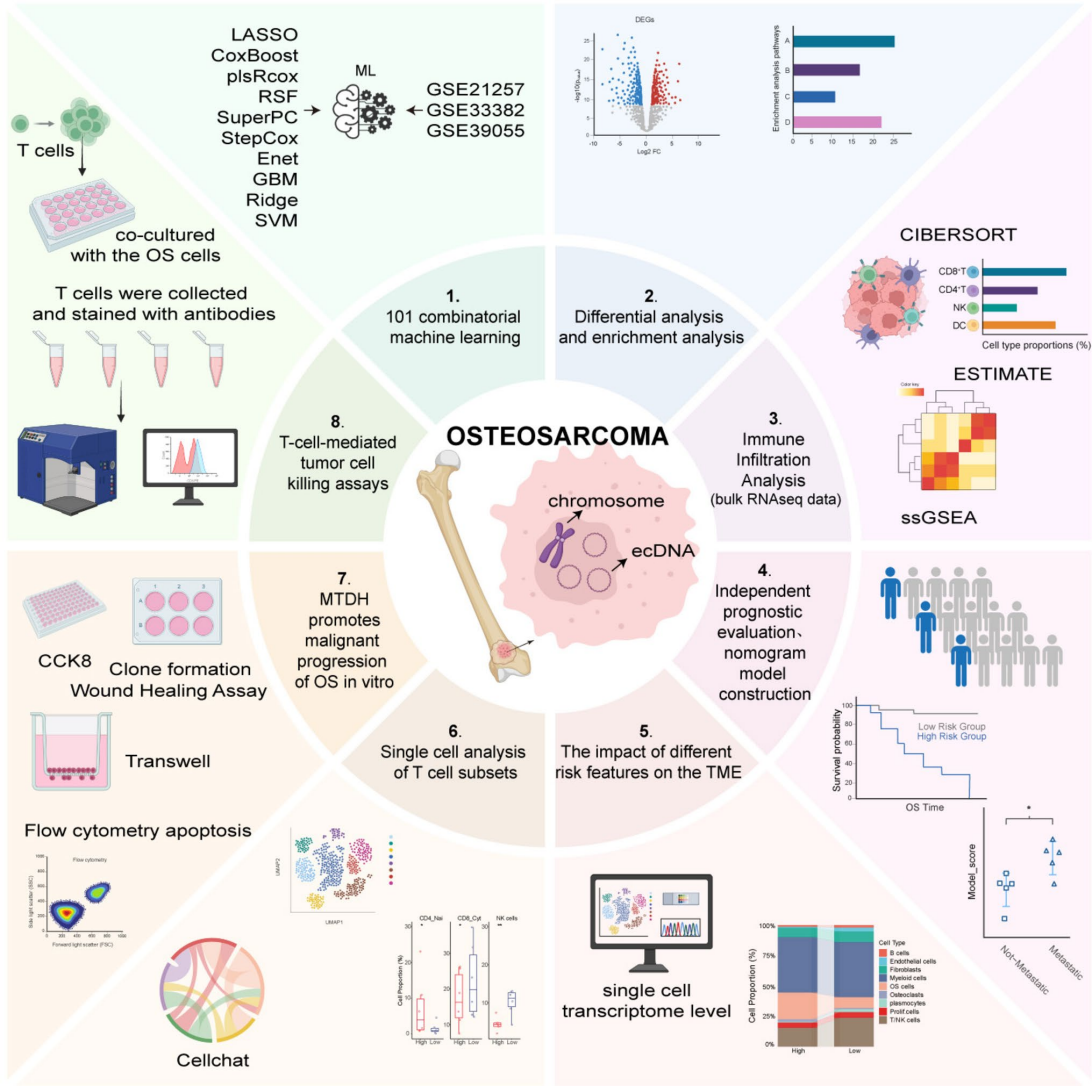
Flowchart of this study

**Fig. 2:**
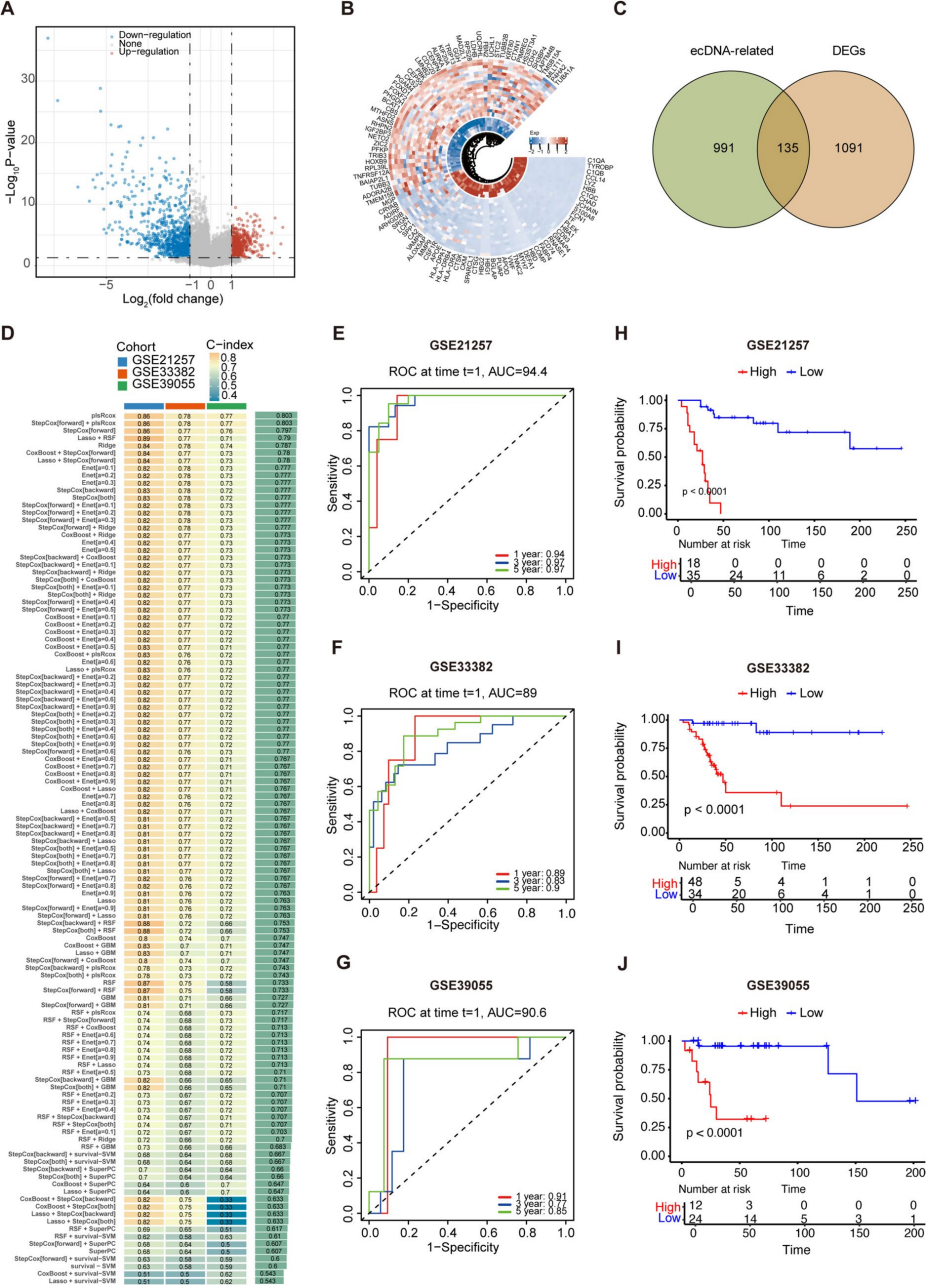
Construction and validation of EGPSM. **A**-**B** Volcano plot **A** and heatmap **B** visualize the DEGs between normal bone tissue and osteosarcoma cell lines in the GSE28424 dataset. **C** Venn diagram illustrating the overlap of osteosarcoma ecDNA-related differentially expressed genes. **D** The C-index of 101 combinations of multiple machine learning models in the training set (GSE21257), testing set (GSE33382), and validation set (GSE39055). **E**–**G** Time-dependent ROC curve analysis of the EGPSM in GSE21257 **E**, GSE33382 **F** and GSE39055 **G**. **H–J** Survival curves for osteosarcoma patients with different risk scores in the GSE21257 **H**, GSE33382 **I**, and GSE39055 **J** cohorts

Figure 1 shows the flowchart of our study. To investigate the role of ecDNA- related genes in osteosarcoma, we first performed differential expression analysis on the GSE28424 dataset to identify DEGs in osteosarcoma (Fig. 2A, B). We then intersected these genes with ecDNA-related genes identified by sequencing in the previous literature[1, 7, 12, 36], resulting in a set of 135 genes (Fig. 2C, Supplementary Table S1). Subsequently, we first performed univariate Cox regression analysis on 135 genes to screen genes associated with prognosis and constructed a prognostic model using 101 machine learning algorithm combinations. The models were built and evaluated in three independent datasets: a training set (GSE21257), a validation set (GSE33382), and an independent validation set (GSE39055). The PlsRcox and stepCox[backward]+plsRcox models achieved the highest mean C-index of 0.803 across the three datasets (Fig. 2D). We selected the stepCox[backward]+plsRcox model to construct the EGPSM due to its superior robustness. Furthermore, we evaluated the prognostic performance of the EGPSM across these datasets. Time dependent ROC (tROC) analysis showed that the 1-, 3-, and 5-year AUCs were 0.94, 0.97, and 0.97, respectively, in the GSE21257 dataset (Fig. 2E). In the GSE33382 dataset, the corresponding AUCs were 0.89, 0.83, and 0.90 (Fig. 2F). In the GSE39055 dataset, the AUCs were 0.91, 0.77, and 0.85, respectively (Fig. 2G). Then, we calculated the risk score for each patient in all datasets based on the EGPSM, and then stratified them into high-risk and low-risk groups based on the median risk scores. Kaplan–Meier (K-M) survival curves demonstrated that patients in the high-risk group had a significantly poorer prognosis in the training set (*P* < 0.0001), validation set (*P* < 0.0001), and independent test set (*P* < 0.0001) (Fig. 2H-J). So far, these results have validated a robust model generated by quantitatively assessing the EGPSM risk state, which may serve as a potential prognostic indicator for predicting treatment benefit in osteosarcoma patients.

### Independent prognostic evaluation and nomogram model construction

**Fig. 3:**
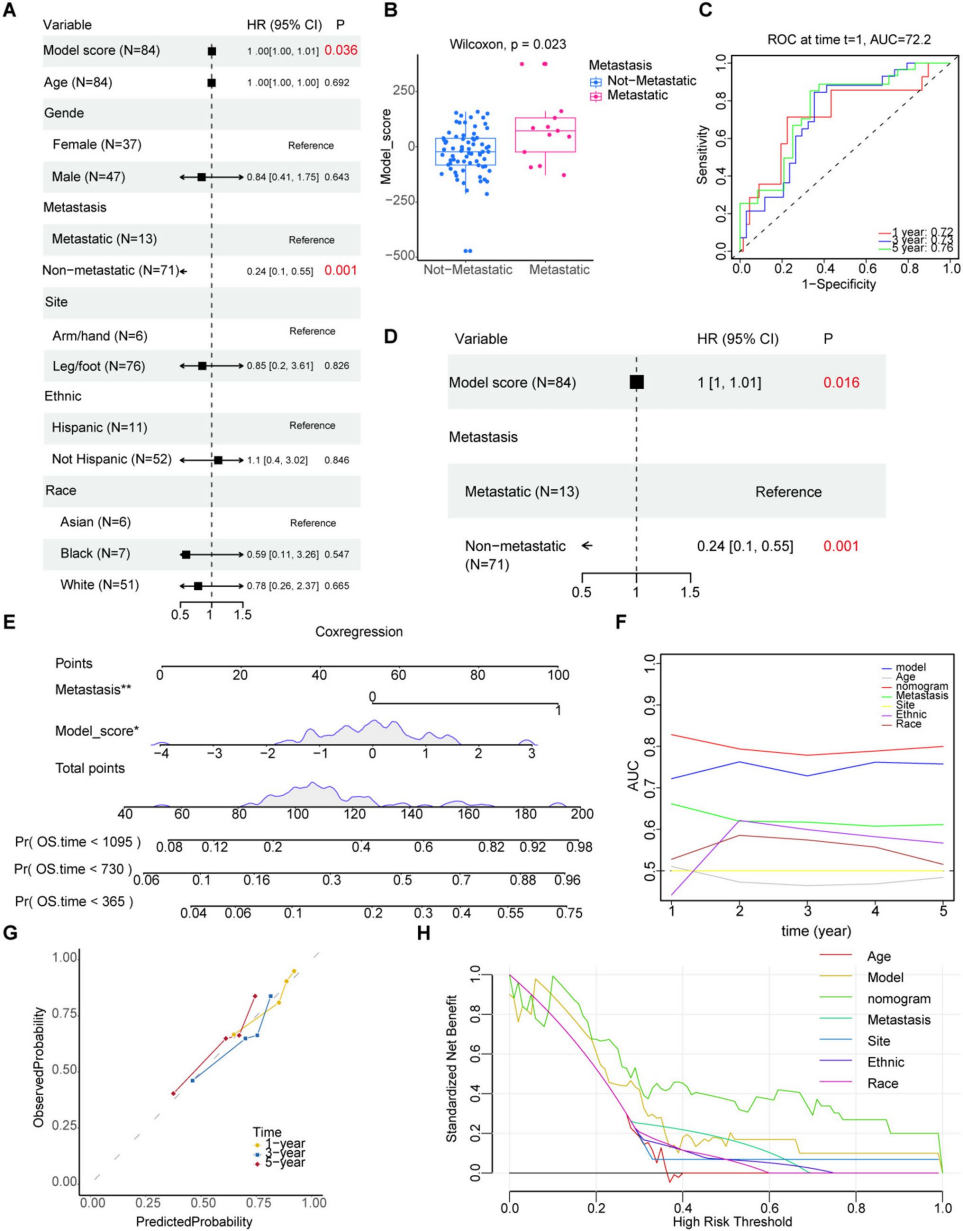
Independent prognostic evaluation and nomogram model construction. **A** Univariate Cox regression analysis of EGPSM and clinical features for predicting osteosarcoma prognosis. **B** Comparison of EGPSM scores in Different Metastatic States. **C** tROC analysis of EGPSM for predicting metastatic status. **D** Muti-cox analysis of EGPSM and clinical features in predicting the osteosarcoma prognosis. **E** Construction of a nomogram model combining EGPSM and clinical features. **F–H** Evaluation of the prognostic nomogram, including tROC analysis **F**, calibration curve **G**, and decision curve analysis **H** *P < 0.05; **P < 0.01; ***P < 0.001

Subsequently, we evaluated the relationship between clinicopathological features and the EGPSM in osteosarcoma patients. Univariate Cox regression analysis was used to determine the factors related to prognosis. The results showed that both EGPSM and metastatic status were significantly associated with patient overall survival (Fig. 3A), with metastatic patients having a higher EGPSM score (Fig. 3B). Time-dependent ROC (tROC) analysis further demonstrated that EGPSM is an effective predictor of metastasis in osteosarcoma patients (Fig. 3C). Next, we performed multivariate Cox regression analysis to evaluate the independent prognostic ability of the variables. The results confirmed that EGPSM and metastatic status are independent predictors of prognosis for osteosarcoma patients (Fig. 3D). To improve the predictive performance of the EGPSM, we constructed a nomogram incorporating the EGPSM score and other key clinicopathological parameters (Fig. 3E). The AUC of the nomogram was significantly higher than that of EGPSM or other parameters alone (Fig. 3F). The nomogram’s calibration curve aligned closely with the ideal diagonal line (Fig. 3G), and a decision curve analysis indicated that the model provides a significant net clinical benefit (Fig. 3H). Collectively, these results underscore the high clinical applicability of the EGPSM in the management of osteosarcoma patients.

### Functional enrichment analysis and immune infiltration analysis

**Fig. 4:**
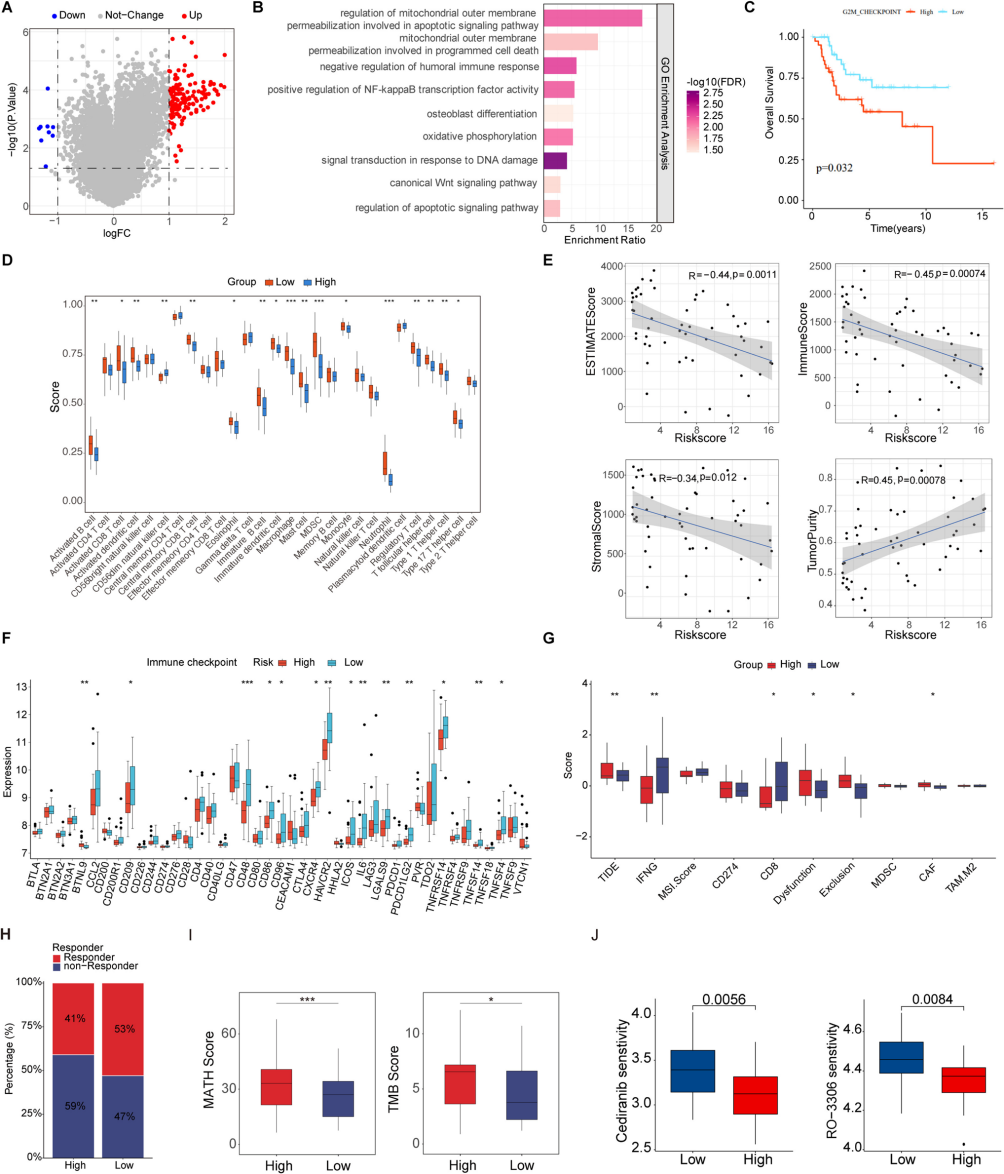
Functional enrichment analysis and immune infiltration analysis. **A** DEGs Between the high and low EGPSM groups in the GSE21257 Dataset. **B** GO enrichment analysis performed on the DEGs. **C** The K-M survival curve revealed a significant correlation between OS and the GSVA score of the G2M CHECKPOINT pathway. **D** Differential immune cell infiltration patterns between the high-risk and low-risk groups. **E** Spearman correlation analysis of ESTIMATE Score, ImmuneScore, StromalScore, Tumor Purity, and risk score. **F** Differential expression of immune checkpoints between the two groups. **G** TIDE prediction of the immunotherapy effectiveness of the two groups. **H** The bar graph depicting the proportion of patients sensitive and resistant to immunotherapy.**I** Box plot showing the difference in Mutant Allele Tumor Heterogeneity (MATH) scores between the high-risk and low-risk groups (left). Distribution of tumor mutational burden (TMB) in the high-risk and low-risk groups(right). **J** Comparison of statistically significant chemotherapy drug IC50 values between the two risk groups. *P < 0.05; **P < 0.01; ***P < 0.001

To elucidate the role of ecDNA-related genes in osteosarcoma, we first identified DEGs between the high- risk and low-risk groups (Fig. 4A). Subsequent GO, KEGG and GSEA enrichment analysis showed that pathways related to tumor progression and immune regulation were significantly enriched (Fig. 4B). These included: regulation of mitochondrial outer membrane permeabilization involved in apoptotic signaling pathway, negative regulation of humoral immune response, positive regulation of NF-κB transcription factor activity, osteoblast differentiation, oxidative phosphorylation, signal transduction in response to DNA damage, and the canonical Wnt signaling pathway. Using GSVA analysis with Hallmark gene sets, we observed the activation of several pathways in the high-risk group, including HALLMARK_DNA_REPAIR, HALLMARK_MYC_TARGETS_V1, and HALLMARK_G2M_CHECKPOINT, with the latter being specifically associated with a poor prognosis (Fig. 4C, Supplementary Figure S1A). Based on these findings, we further evaluated immune infiltration between the high-risk and low-risk groups. Our analysis of immune cell abundance revealed a significant reduction in several tumor-killing immune cell types in the high- risk group, including activated B cells, activated and immune dendritic cells, mast cells, and neutrophils (Fig. 4D). Consistent with this, immune infiltration analysis showed that patients in the high-risk group had a higher tumor purity score but lower ESTIMATE, immune, and stromal scores (Fig. 4E, Supplementary Figure S1B-E). Furthermore, we observed the downregulation of several key checkpoint genes in the high- risk group (Fig. 4F). We further explored the differential expression of immune checkpoints, immunomodulatory factors, and MHC molecules in different groups to predict the response of immunotherapy in different osteosarcoma strata. As a key prognostic indicator of response to immunotherapy, several immune checkpoints were significantly upregulated in low-risk patients, including CD209, CD48, CD86, CD96, CXCR4, HAVCR2, ICOS, IL6, LGALS9, PDCD1LG2, and TNFRSF14 (Fig. 4F). Furthermore, we observed a significant inverse correlation between the risk score and the expression levels of these immune checkpoints (Supplementary Figure S2A). Additionally, most MHC molecules, such as HLA-DMA, HLA-DMB, HLA-DOA, HLA-DPB1, HLA-DPB2, HLA-DQA1 and HLA-E, showed considerably high expression levels in the low-risk group (Supplementary Figure S2B). The low-risk group also had elevated levels of most chemokines, interleukins, interferons, receptors, and other cytokines (Supplementary Figure S2C). Analysis based on the TIDE algorithm revealed that the high-risk group exhibited significantly higher TIDE scores, as well as elevated Dysfunction, Exclusion, and CAF scores (Fig. 4G). These findings suggest that the high-risk group is characterized by a dual mechanism of immune evasion: on one hand, the infiltrating T cells manifest profound functional exhaustion; on the other hand, high levels of CAF infiltration likely construct a physical barrier that restricts the infiltration of effector immune cells. Furthermore, subsequent analysis demonstrated a higher proportion of responders in the low-risk group compared to the high-risk group (53 vs. 41%) (Fig. 4H), indicating a propensity for immune surveillance evasion in high-risk group and a less favorable response to immune checkpoint inhibitors. Generally, genetic mutations can influence tumor progression and therapeutic response by modulating the molecular genetic profiles of tumor cells. Our comprehensive overview of the TCGA mutation data indicated that missense mutations were the most prevalent category within the variant classification, with single nucleotide polymorphisms (SNPs) being the most frequent variant type. Additionally, the median number of variants per sample was observed to be 43(Supplementary Fig. 3B). Specifically, by integrating mutation data with the high- and low-risk groups, we analyzed the top 15 mutated genes and constructed corresponding oncoplots (waterfall plots). As illustrated, the mutation frequencies of the top 15 genes in the high-risk and low-risk groups were 73.33% and 69.83%, respectively (Supplementary Fig. 3A). Among all identified genes, TP53 exhibited the highest mutation frequency across both risk groups. Intratumor heterogeneity (ITH) refers to the genetic diversity existing among different cell subpopulations within a tumor, typically arising from the accumulation of mutations during tumor progression. ITH is closely associated with rapid tumor progression, metastasis, and chemoresistance, which frequently lead to treatment failure. To quantify the ITH in patients with osteosarcoma, we applied the MATH algorithm, where a higher MATH score represents a higher degree of ITH. Our analysis revealed that the MATH scores in the high-risk group were significantly higher than those in the low-risk group (Fig. 4I). Furthermore, we analyzed the TMB and found that TMB values in the high-risk group were concentrated in a higher range, indicating a greater accumulation of somatic mutations in this group (Fig. 4I). These findings collectively suggest that the low-risk group has enhanced sensitivity to immunomodulatory factors and better response to immunotherapy. Finally, we evaluated the chemotherapeutic response by calculating the half-maximal inhibitory concentration (IC50) values for over 100 chemotherapeutic drugs. The results indicated significant differences in drug sensitivity between the two groups, with nine chemotherapeutic agents, including Cediranib and RO-3306, showing higher sensitivity in the high-risk group (Fig. 4J, Supplementary Figure S1F). These findings collectively reveal that the efficacy of immunotherapy and chemotherapy varies according to EGPSM stratum, with lower-risk patients having better outcomes with immunotherapy.

### Based on single-cell transcriptomics analysis of the EGPSM

**Fig. 5:**
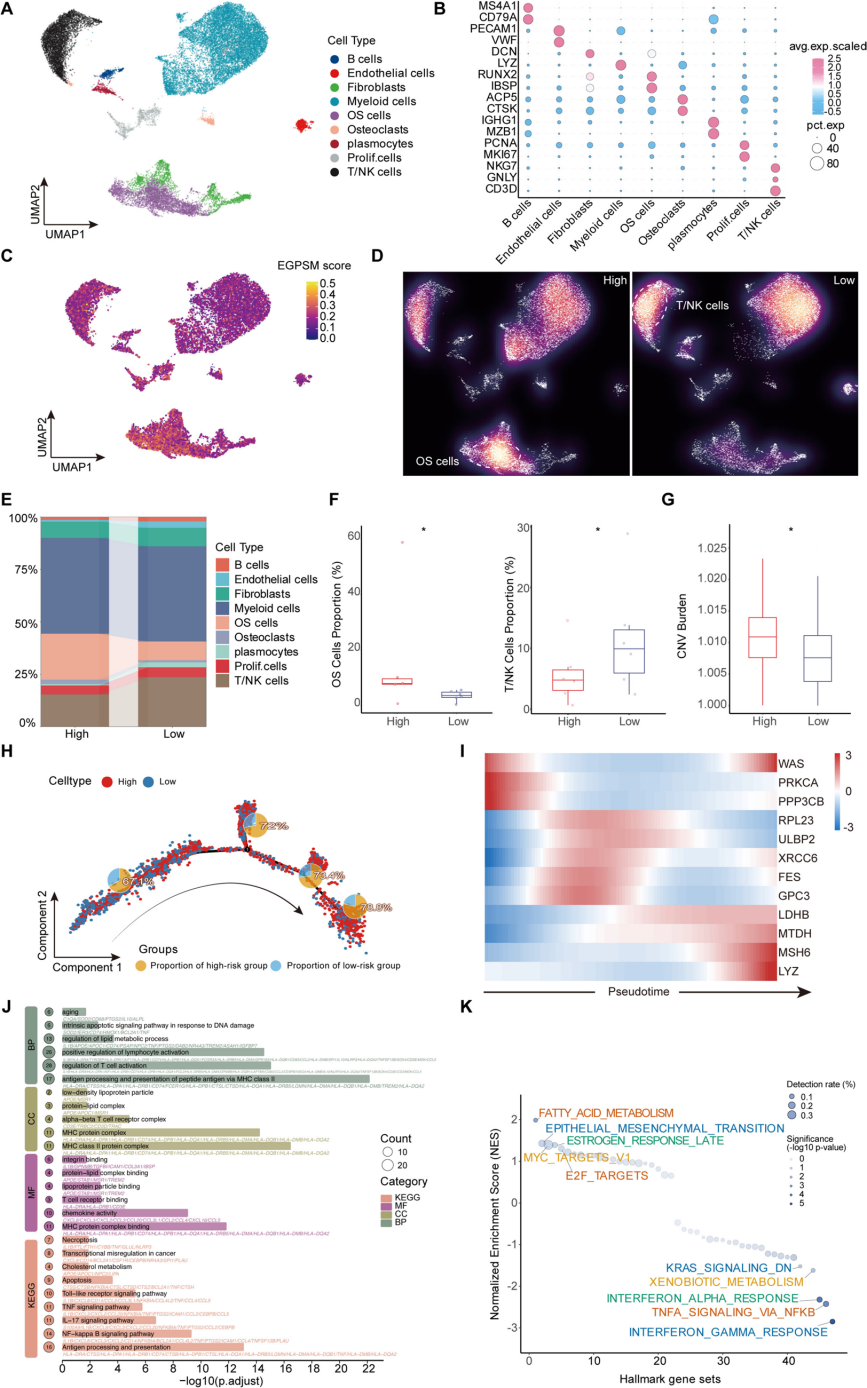
Based on scRNA-seq analysis of the EGPSM. **A** UMAP dimensionality reduction plot of the scRNA-seq profiles of osteosarcoma. **B** Bubble plot showing the marker genes utilized for cell subpopulation annotation. Dot size is proportional to the fraction of cells expressing specific genes, and color intensity corresponds to the relative expression level of the selected genes. **C** A UMAP plot showing EGPSM score for different cell subtypes. **D** Galaxy plots showing the distribution density of various cell types in the UMAP space for the high-risk (left) and low-risk (right) groups. Low density is represented by cooler colors, while high density is represented by warmer colors. **E** A stacked bar chart of cell proportions shows the distribution of cells across different risk score groups. **F** The proportions of two cell subpopulations were compared between the high and low risk groups. **G** Differences in inferCNV score among different risk groups. **H** Pseudotime trajectory analysis of osteosarcoma cells and the corresponding percentage of high EGPSM score cells. **I** Pseudotime heatmap illustrating the differentiation trajectory of EGPSM genes. **J–K** The GO/KEEG analysis **J** and GSEA analysis **K** of DEGs between cells in two EGPSM score groups. *P* < 0.05

To better elucidate how EGPSM shapes the osteosarcoma TME, we conducted a series of single cell transcriptomic analyses. We performed scRNA-seq analysis on six patient samples retrieved from the GEO database (accession number GSE162454) to further explore the role of the EGPSM in osteosarcoma. After stringent quality control filtering, we used the “harmony” algorithm for batch correction and performed dimensionality reduction and clustering with PCA and UMAP (Supplementary Figure S4A). Based on previously reported cell markers, we identified 10 distinct cell subpopulations: B cells, Endothelial cells, Fibroblasts, Myeloid cells, Osteoblastic cells, Osteoclasts, Plasmocytes, Prolif.cells, Prolif. Osteoblastic cells, and T/NK cells (Fig. 5A). The rationality of cell annotation was further verified by bubble plots (Fig. 5B). To facilitate further analysis, we used the “AddModuleScore” package to score all cells based on the relevant genes from our EGPSM (Fig. 5C). Based on the AddModuleScore results, cells were stratified into a high EGPSM score group and a low EGPSM score group using the median score as a cutoff (Supplementary Figure S4D). Cell component analysis showed that the high EGPSM score group had a higher proportion of osteosarcoma cells and osteoclasts, while the ratio of T/NK cells was decreased (Fig. 5E, Supplementary Figure S4B). Furthermore, a statistical analysis of cell proportions within samples showed an increase in osteosarcoma cells and a decrease in T/NK and endothelial cells in the high EGPSM score group (Fig. 5F, Supplementary Figure S4E). Given our previous finding from GO enrichment analysis of the osteoblast differentiation pathway, we performed a pseudo-temporal trajectory analysis on the osteosarcoma cells to further investigate their differentiation dynamics. Interestingly, as osteosarcoma cells differentiate, high risk malignant tumor cells gradually become enriched, and the EGPSM score shows a progressive increasing trend (Fig. 5H-I, Supplementary Figure S4F). Additionally, the high EGPSM score group exhibited significantly elevated CNV burden (Fig. 5G, Supplementary Figure S4C), indicating a higher degree of genomic instability that potentially correlates with the presence of ecDNA during the malignant progression of osteosarcoma. We also analyzed the DEGs between the two groups and performed enrichment analysis, which further revealed numerous differences in oncogenic and immune-related signaling pathways (Fig. 5J-K), consistent with our bulk RNAseq analysis results. In addition, metabolic analysis showed distinct metabolic features between the two groups (Supplementary Figure S4G). These findings collectively suggest that EGPSM score promotes the malignant progression of tumor cells and contributes to the shaping of a “cold” tumor microenvironment.

### ScRNAseq analysis showed different characteristics of T cells related to the EGPSM score

**Fig. 6:**
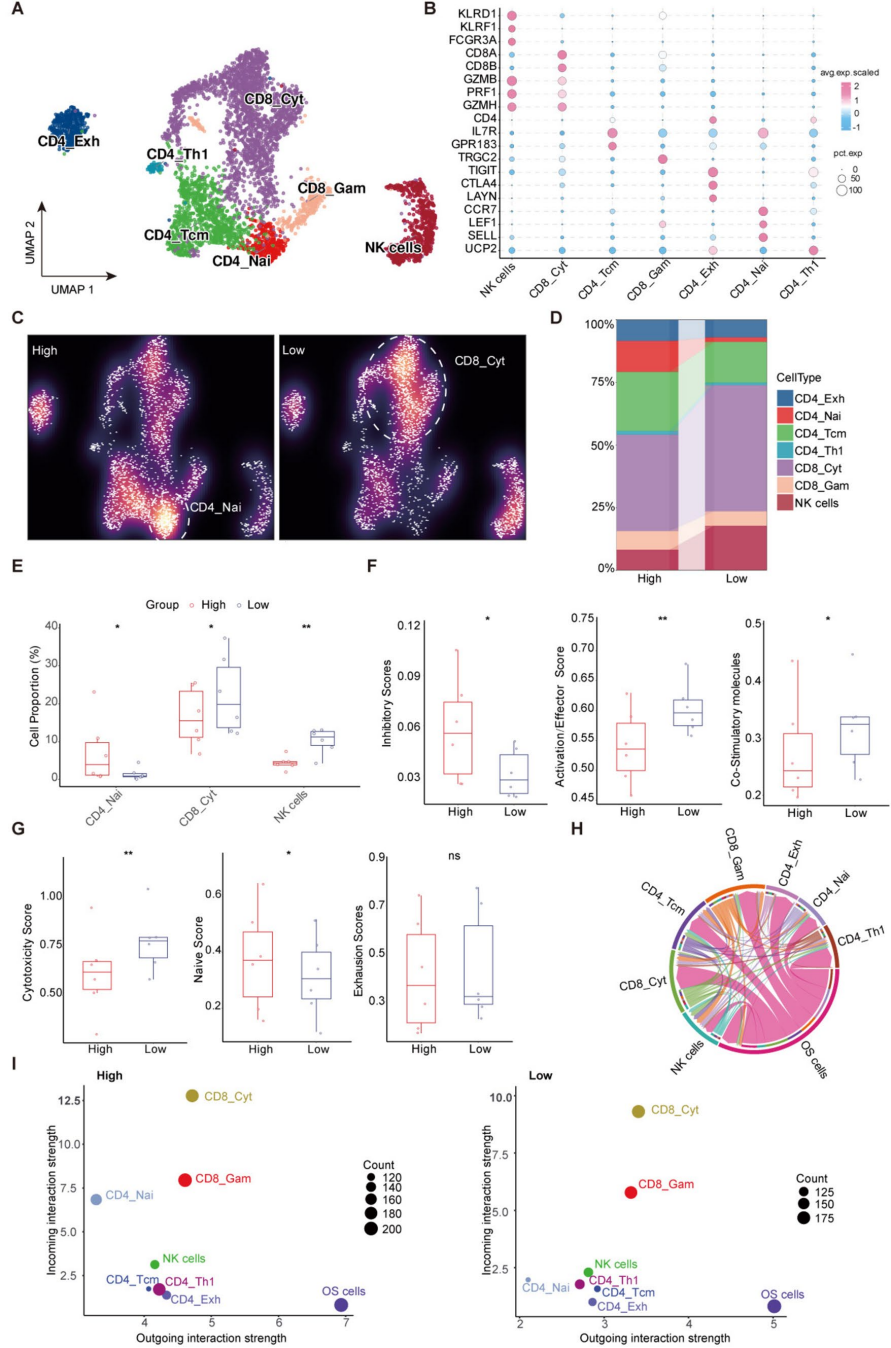
ScRNA-seq analysis showed different characteristics of T cells related to the EGPSM score. **A** UMAP plots of the scRNA-seq profiles of T/NK cells. **B** Bubble plot showing the marker genes utilized for cell subpopulation annotation. Dot size is proportional to the fraction of cells expressing specific genes, and color intensity corresponds to the relative expression level of the selected genes. **C** Galaxy plots showing the distribution density of various cell types in the UMAP space for the high-risk (left) and low-risk (right) groups. Low density is represented by cooler colors, while high density is represented by warmer colors. **D** A stacked bar chart of cell proportions shows the distribution of cells across different EGPSM score groups. **E** The proportions of two cell subpopulations were compared between the high and low risk score groups. **F–G** Box plots comparing functional scores among different risk score groups. Metrics evaluated include Inhibitory levels, activation/effector potential, co-stimulatory molecules, cytotoxicity levels, naive phenotype, and exhaustion levels. **H-I** The number and strength of cell–cell interaction relationships between osteosarcoma cells and T/NK cell subsets. *P < 0.05; **P < 0.01; ***P < 0.001

Given the critical role of T cells in anti-tumor immunity, we performed an unsupervised clustering subpopulation analysis on T/NK cells to further investigate their characteristics in different groups. We categorized cell types into CD4_Exh, CD4_Th1, CD4_Tcm, CD4_Nai, CD8_Cyt, CD8_Gam, and NK cells (Fig. 6A), with validation through marker gene expression (Fig. 6B). Subsequent cell composition analysis revealed a reduced proportion of CD8_Cyt and NK cells and a higher proportion of CD4_Nai cells in the high EGPSM score group (Fig. 6C-D, Supplementary Figure S5A-B). Interestingly, analysis of T cell-related functional state scores between the different groups revealed that the high EGPSM score group exhibited enhanced inhibitory and naive-like characteristics, while displaying fewer activation, stimulation, and cytotoxicity features. No significant difference in the exhausted state was observed between the two groups (Fig. 6F-G). This indicates that EGPSM score plays a crucial regulatory role in shaping the immune landscape of osteosarcoma. Specifically, our study suggests that the high EGPSM score group defines a tumor immune microenvironment primarily characterized by an "immune-cold" state. As osteosarcoma cells showed an imbalanced distribution between the different score groups, we speculated that they might contribute to shaping this “cold” tumor microenvironment. We then performed a CellChat analysis and found a strong interaction between osteosarcoma cells and both CD8_Cyt and CD4_Nai cells (Fig. 6E). Notably, the high EGPSM score group showed stronger interaction levels between osteosarcoma cells and CD8_Cyt and CD4_Nai cells (Fig. 6G, Supplementary Figure S5C). Finally, a differential signaling pathway analysis between the two groups revealed a significant enrichment of the TIGIT pathway in the high EGPSM score group (Supplementary Figure S5D). This provides a mechanistic explanation for immune exclusion: the tumor cells utilize potent TIGIT-mediated inhibitory signaling to intercept and suppress T cells at an early stage, thereby preventing effective infiltration and maintaining the "immune-cold" state.

### MTDH promotes tumor progression in osteosarcoma

**Fig. 7:**
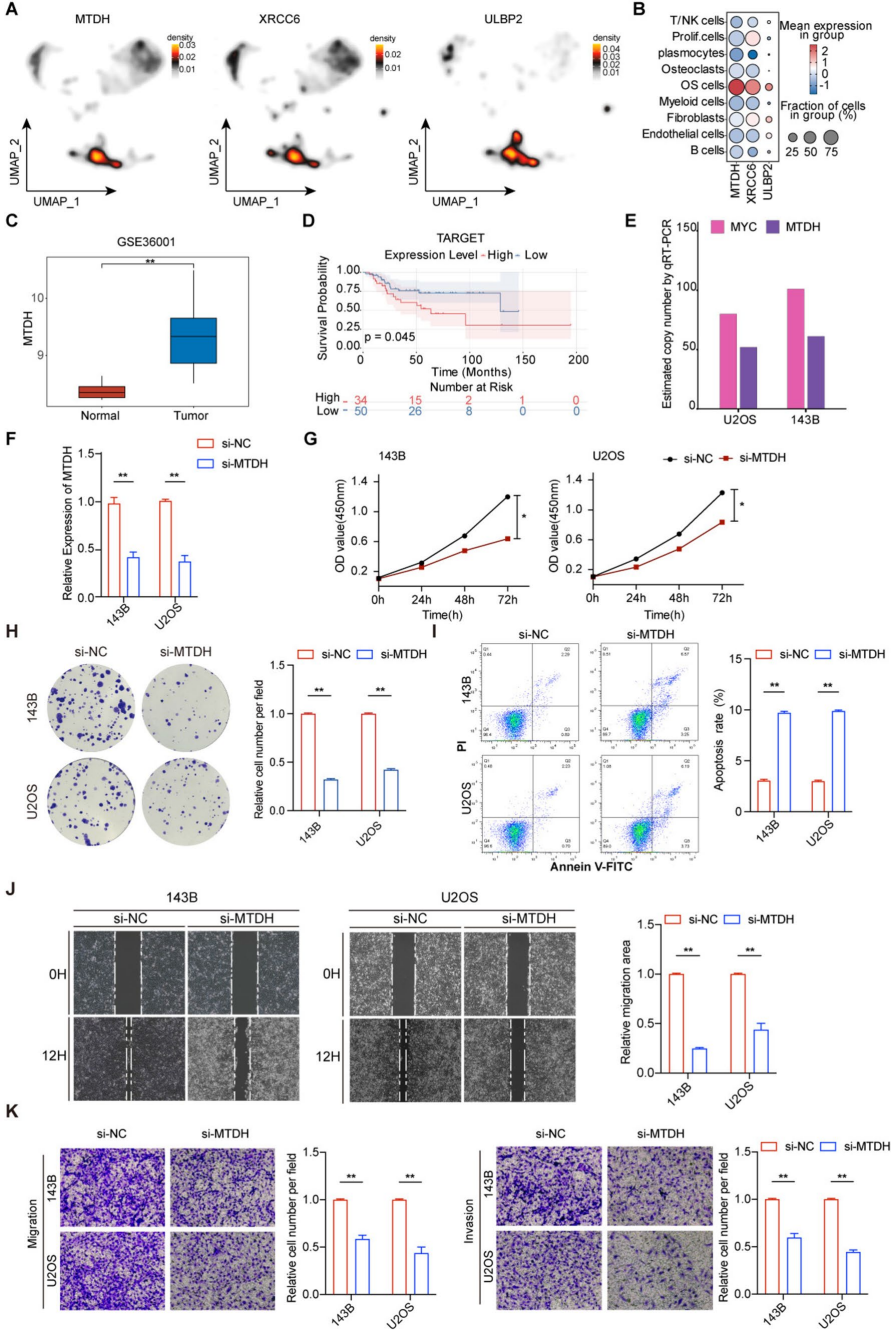
MTDH promotes tumor progression in osteosarcoma. **A** UMAP plot showing gene expression density. **B** Bubble plot showing the expression of three genes (MTDH, XRCC6, and ULBP2) across different cell types. Dot size is proportional to the fraction of cells expressing specific genes, and color intensity corresponds to the relative expression level of the selected genes. **C** Comparison of MTDH expression between osteosarcoma and normal tissues in the GSE36001 dataset. **D** Overall survival differences between osteosarcoma patients with high and low MTDH expression in the TARGET database. **E** Estimated gene copy numbers of MYC and MTDH in 143B and U2OS cells by qRT-PCR. **F** The efficiency of MTDH knockdown in 143B and U2OS cells was detected by qRT-PCR assays, respectively. **G** CCK-8 assay tested the effect of MTDH on the proliferation ability of 143B and U2OS cells. **H** Colony formation assay and quantitative analysis were performed to test the effect of MTDH knockdown on osteosarcoma cellline proliferation. **I** Flow cytometry quantified the percentage of apoptotic cells after the specified intervention. **J** Wound healing assay was performed to evaluate the effect of MTDH on the migration ability of 143B and U2OS cells. **K** The impact of MTDH on cell migration and invasion was investigated using transwell assays in 143B and U2OS cells. *P < 0.05; **P < 0.01

To identify potential EGPSM-associated therapeutic targets in osteosarcoma, we first examined the cell specific expression levels of EGPSM genes using our scRNAseq data. We subsequently found that MTDH, XRCC6, and ULBP2 were specifically highly expressed in osteosarcoma cells (Fig. 7A, Supplementary Figure S6A). A bubble plot visualizing the expression levels of these three genes showed that MTDH exhibited the highest expression and cell-specificity in osteosarcoma cells, highlighting its potential as a therapeutic target (Fig. 7B). Subsequent analysis of the GSE36001 dataset confirmed that MTDH was significantly overexpressed in osteosarcoma, and its high expression was found to be associated with poor prognosis across multiple datasets (Fig. 7C-D, Supplementary Figure S6B). Furthermore, Quantitative real-time PCR (qPCR) analysis revealed that compared to normal osteoblast cells (hfob1.19), MTDH expression was significantly upregulated in various osteosarcoma cell lines (Supplementary Figure S6C). To further elucidate the oncogenic function of MTDH, we conducted a series of in vitro experiments. The qRT-PCR was assessed to evaluate the copy numbers of MTDH and MYC, which were found to be aberrantly elevated, consistent with critical features of ecDNA (Fig. 7E). We then performed siRNA mediated knockdown to silence MTDH in 143B and U2OS cells and validated the knockdown efficiency (Fig. 7F). CCK-8 and colony formation assays showed that MTDH knockdown significantly inhibited the proliferation of both 143B and U2OS cells (Fig. 7G-H). A flow cytometry-based apoptosis assay demonstrated that MTDH knockdown promoted cellular apoptosis (Fig. 7I). Furthermore, wound healing and Transwell assays showed that MTDH knockdown reduced the migration and invasion capabilities of the osteosarcoma cells (Fig. 7J-K). Conversely, we established stable overexpression cell lines in 143B and U2OS cells, and the overexpression efficiency was validated at both mRNA and protein levels (Supplementary Fig. 7A-B). CCK-8 and colony formation assays revealed that MTDH overexpression significantly promoted the proliferation and tumorigenic potential of both osteosarcoma cell lines (Supplementary Fig. 7C-D). Flow cytometry analysis indicated that MTDH overexpression led to a reduction in the apoptotic rate compared to the control group (Supplementary Fig. 7E). Furthermore, wound healing and Transwell assays demonstrated that elevated MTDH expression markedly enhanced the migratory and invasive capacities of 143B and U2OS cells (Supplementary Fig. 7G-H). These findings collectively indicate that MTDH promotes the malignant progression of osteosarcoma and thus represents a potential therapeutic target for osteosarcoma patients.

### Disruption of MTDH enhances CD8 + T-cell-mediated antitumor immunity

**Fig. 8:**
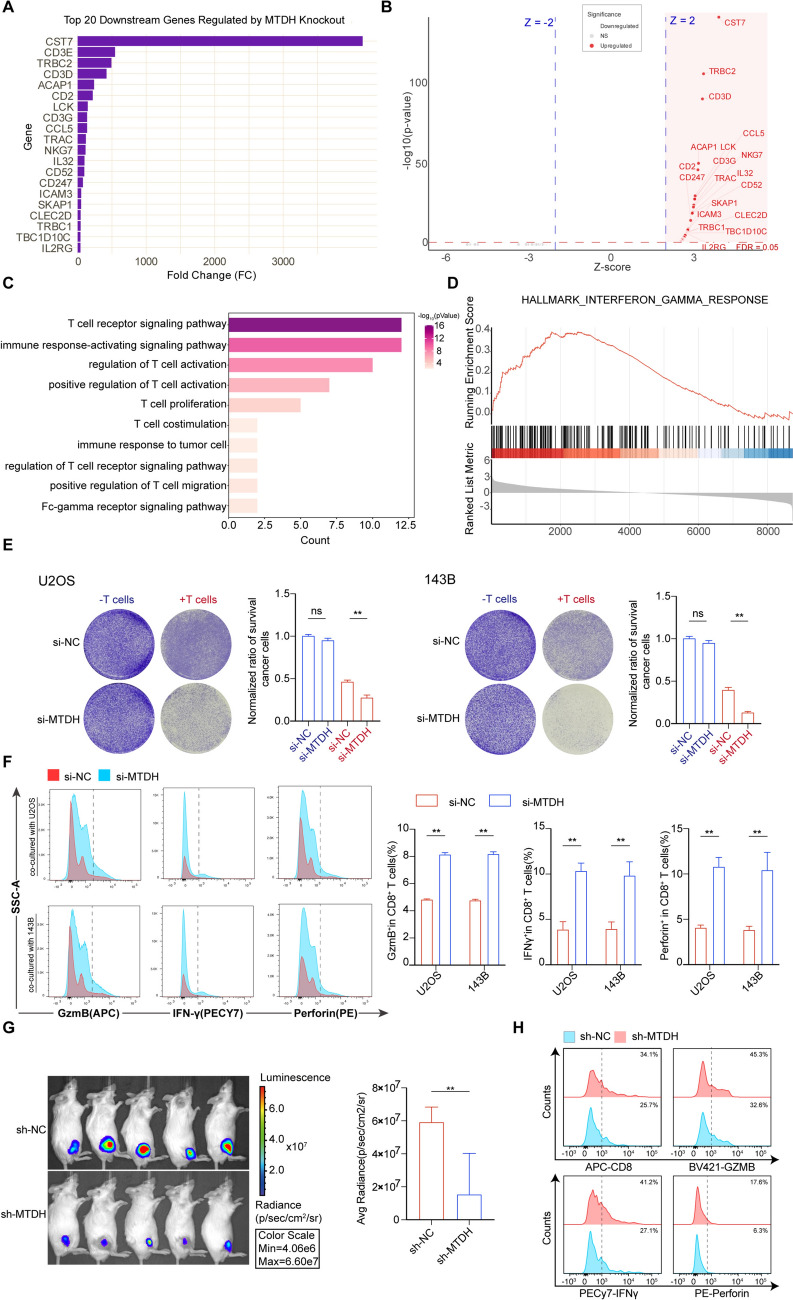
MTDH disruption promotes CD8.^+^ T cell-mediated antitumor immunity suppressing OS cells growth. **A** Bar plot showing significantly affected genes upon virtual knockout of MTDH. **B** Volcano plot showing significantly dysregulated genes following virtual knockout of MTDH. **C**-**D** GO enrichment analysis **C** and GSEA enrichment analysis **D** after MTDH virtual knockout. **E** A T cell-mediated tumor cell killing assay was performed by co-culturing CD8⁺ T cells with 143B or U2OS cells for 3 days. The left panel shows representative images of surviving tumor cells stained with crystal violet, and the right panel presents the quantification of staining intensity of the surviving cells. **F** CD8⁺ T cells were co-cultured for 48 h with 143B and U2OS cells that had been transfected with either control or siMTDH. The fractions of GzmB⁺, IFN-*γ*⁺ and Perforin⁺ cells within the CD8⁺ T cell population were then quantified by flow cytometry. **G** Representative bioluminescence imaging (left) and quantification of luciferase signals (right) in mice injected with sh-NC or sh-MTDH K7M2 cells into the tibia, showing reduced tumor burden four weeks post-injection upon MTDH knockdown. **H** Flow cytometry analysis of tumors harvested from the tibial orthotopic model. The percentages of total CD8⁺ T cells (top left), as well as the percentages of Granzyme B⁺ (top right), IFN-*γ*⁺ (bottom left), and Perforin⁺ (bottom right) cells within the CD8⁺ T cell population, were quantified, showing enhanced infiltration and function of CD8⁺ T cells in the sh-MTDH group.*P < 0.05; **P < 0.01

To further investigate MTDH’s role in shaping the TME, we used scRNA-seq data to simulate MTDH knockout and analyzed the resulting transcriptional changes (Supplementary Figure S9A). We then visualized the genomic changes induced by this knockdown, as shown in Figs. 8A and 8B. We also performed GO and KEGG enrichment analyses to further explore MTDH’s influence on the osteosarcoma microenvironment (Fig. 8C-D, Supplementary Figure S9B-C).Remarkably, the MTDH knockdown led to the activation of several key pathways, including the T-cell receptor signaling pathway, immune response-activating signaling pathway, regulation of T-cell activation, positive regulation of T-cell activation, T-cell proliferation, T-cell costimulation, and the Fc-gamma receptor signaling pathway. GSEA enrichment analysis further identified the interinterferon gamma response pathway, which is strongly associated with immune activation. These findings suggest that MTDH may drive the formation of an "immune-cold" microenvironment in osteosarcoma, primarily characterized by immune exclusion and impaired T-cell infiltration. Our prior immune infiltration analysis had already revealed a negative correlation between MTDH and CD8⁺ T cells (Supplementary Figure S9D). As key effector cells in the immune system, CD8⁺ T cells-particularly the CD8_Cyt are primarily responsible for killing tumor cells. To validate these findings, we conducted subsequent in vitro T-cell killing assays. We first silenced MTDH in 143B and U2OS cells using siRNAs and then co-cultured them with activated CD8⁺ T cells. We found that the CD8⁺ T cells co-cultured with the siMTDH-transfected tumor cells exhibited a significantly enhanced tumor-killing effect compared to the control group (Fig. 8E). Flow cytometry experiments also revealed a significant increase in the levels of granzyme B (GzmB), IFN-*γ*, and perforin (Fig. 8F), which are all crucial for CD8⁺ T cell killing activity. Subsequently, we co-cultured activated CD8⁺ T cells with the established MTDH-stable overexpression cell lines of 143B and U2OS.We observed that the tumor-killing efficacy of CD8⁺ T cells co-cultured with MTDH-overexpressing tumor cells was significantly reduced compared to the control group (Supplementary Fig. 8A). Furthermore, flow cytometry analysis revealed a marked decrease in the expression levels of GzmB, IFN-γ, and perforin in the CD8⁺ T cells (Supplementary Fig. 8B), all of which are crucial for CD8⁺ T cell-mediated cytotoxicity. To investigate the role of MTDH in tumor growth and the immune microenvironment in vivo, we established an orthotopic mouse model by injecting K7M2 osteosarcoma cells (stably expressing luciferase) into the tibia of BALB/c mice. Mice were divided into two groups: control (sh-NC) and MTDH-knockdown (sh-MTDH). Bioluminescence imaging (BLI) performed four weeks post-injection showed that silencing MTDH significantly reduced the tumor burden compared to the control group (Fig. 8G). Subsequent flow cytometry analysis of the harvested tumors revealed that MTDH knockdown increased the infiltration of CD8⁺ T cells within the tumor microenvironment. Furthermore, CD8⁺ T cells from the sh-MTDH tumors exhibited significantly enhanced functional activity, as evidenced by the elevated percentages of GzmB⁺, IFN-*γ*⁺, and Perforin⁺ cells compared to the control group (Fig. 8H), Supplementary Fig. 7C). These results suggest that MTDH inhibits CD8⁺T cell-mediated antitumor immunity, thereby promoting osteosarcoma growth.

## Discussion

Osteosarcoma remains a highly aggressive malignancy with a poor clinical prognosis. Although limited progress has been made with conventional radio- and chemotherapy as well as immunotherapy, patient survival rates have plateaued, a challenge that is especially prominent in patients with recurrent or metastatic disease [7, 19, 37]. Metastatic dissemination, chemoresistance, and disease relapse converge to drive poor outcomes, yet the underlying pathogenic mechanisms remain incompletely elucidated [38]. In this study, we constructed a new prognosis model associated with immune stratification by combining machine learning, and the model exhibited good discrimination and calibration abilities. More importantly, we found that MTDH promotes the malignant progression of osteosarcoma by shaping the formation of the osteosarcoma “immune-cold” microenvironment.

Widespread research indicates that ecDNA is considered a major contributor to cancer pathogenesis found in most cancer types, and it is associated with poor outcomes[39, 40]. However, its role in osteosarcoma remains unclear. Limited studies indicate that when DNA sustains multiple small-scale breaks, fragments failing to reintegrate into derivative chromosomes can, instead of being lost, self-ligate to form ecDNA structures that potently amplify oncogenes. To the best of our knowledge, only one study has reported that in parosteal osteosarcomas co-amplification of MDM2 and CDK4 was mapped to ecDNA events in every case [41]. A large-scale analysis of 14,778 patients across 39 tumor types from the 100,000 Genomes Project demonstrated that ecDNA correlates with advanced tumor stage, therapeutic resistance, metastasis, and poor clinical outcome [6, 42]. In agreement with these findings, our data show that the EGPSM high-risk group exhibits inferior prognosis and is enriched for malignant osteoblasts, indicating that ecDNA-related transcriptional programs may underlie aggressive tumor phenotypes in osteosarcoma. Pseudotime analysis further revealed a progressive increase in EGPSM score along the differentiation trajectory of osteoblastic tumor cells, suggesting an escalation of genomic instability. This trend is potentially linked to the gradual accumulation of ecDNA-related features during tumor evolution, as reported in previous literature[7, 39]. We also observed significantly elevated inferCNV scores in the high-risk group, suggesting enhanced genomic instability. This observation is consistent with the report by Lv et al., which demonstrated that ecDNA^+^ tumor cells carry higher CNV burdens than ecDNA^−^cells. These findings support the view that the non-Mendelian inheritance and high copy number of ecDNA contribute to chromosomal fragmentation and rearrangement[41, 42]. Functional enrichment analysis revealed alterations in lipid metabolism, DNA damage response, NF-*κ*B signaling, and apoptosis, which are established hallmarks of osteosarcoma progression [43–46].

Emerging evidence suggests that ecDNA not only drives genomic instability but also promotes immune evasion. Approximately 34% of ecDNA⁺ tumors harbor amplification of immune regulatory genes, consequently leading to diminished T-cell infiltration. [6, 7, 47]. Zhang et al. further showed that ecMYC⁺ lung cancer cell lines suppress antigen presentation and cytokine signaling despite active proliferation, indicating a link between ecDNA and “immune-cold” transcriptional states [47]. Consistent with this, our bulk RNA-seq immune module analysis showed that EGPSM-defined high-risk patients exhibit reduced immune infiltration and higher tumor purity, along with depletion of CD8⁺ T cells, NK cells, dendritic cells, and neutrophils. Moreover, T cells in the high-risk group displayed an inactive or quiescent profile, characterized by a predominance of naive or resting states rather than cytotoxic activation. These observations concord with prior evidence that ecDNA-rich tumors are associated with impaired anti-tumor immunity and immune exclusion [6].

It has been reported that ecDNA also influences response to immunotherapy by promoting immune escape [48, 49]. Clinical observations reveal that tumors with a higher ecDNA burden often show reduced benefit from immune checkpoint inhibitors (ICIs), suggesting ecDNA as a potential negative predictive biomarker [1]. Considering that only a minority of patients with advanced osteosarcoma respond to immunotherapy [50–52], identifying molecular determinants of immunotherapy sensitivity is of clinical relevance. Earlier studies reported that patients with ecDNA-driven tumors display lower ICI response rates due to transcriptional suppression of immune effector pathways [6, 53]. Consistent with this, our comparative analysis revealed significantly higher immune checkpoint expression in the low-risk group defined by EGPSM, implying that these patients retain an immune-active tumor microenvironment and may derive greater benefit from ICIs. In contrast, the high-risk group showed marked downregulation of antigen presentation genes, including multiple HLA family members, suggesting impaired tumor antigen visibility and reduced responsiveness to immunotherapy. These findings implicate defective antigen presentation as a key mechanism of ecDNA-associated immune tolerance and suggest that combination strategies with immunostimulatory agents may be required to restore immune activation in high-risk patients. Our observations are in line with Lv et al., who reported that ecDNA⁺ malignant cells exhibit reduced MHC-I expression and immune evasion phenotypes [7]. Similarly, another transcriptomic analysis demonstrated that ecDNA is associated with diminished immune infiltration, reduced cytotoxic T-cell activity, and lower expression of MHC-I and MHC-II molecules [54]. Together, these findings indicate that ecDNA-related genes reflect an “immune-cold” state and may serve as molecular indicators of immunotherapy resistance. Accordingly, the EGPSM may help define ecDNA-associated immune subtypes in osteosarcoma and guide personalized immunotherapy decisions.

In this study, we identified MTDH as a malignancy-related gene in osteosarcoma and our findings are consistent with recent reports showing that MTDH orchestrates oncogenic signaling, epithelial mesenchymal transition (EMT), and metastatic dissemination in aggressive sarcomas [55]. Previous studies demonstrated that MTDH enhances invasiveness and survival through activation of NF-κB, PI3K/AKT, and MAPK signaling, supporting its role as a central driver of tumor progression across cancers [56]. In line with these findings, MTDH has been mechanistically validated as a multifunctional oncogene in breast and hepatocellular carcinoma, where it promotes metastatic colonization and chemoresistance and is linked to maintenance of stem-like phenotypes[57, 58]. Importantly, recent breakthroughs revealed that MTDH forms a protein complex with SND1 that suppresses antigen presentation by destabilizing TAP1/2 mRNA, thereby facilitating immune evasion; disruption of this complex restores CD8⁺ T-cell cytotoxicity and synergizes with PD-1 blockade in vivo [59, 60]. These immune-related functions align with our observation that MTDH expression is associated with reduced cytotoxic T-cell activity in osteosarcoma, suggesting that MTDH contributes to shaping an immunosuppressed tumor microenvironment. Emerging evidence has also connected MTDH to genomic instability, and since MTDH is located at 8q22.1, a region prone to amplification, it may participate in ecDNA-related oncogene activation, a process known to confer transcriptional plasticity and immune escape in aggressive tumors[6, 48]. Collectively, current evidence from multiple tumor types indicates that MTDH functions not only as a malignant driver but also as an immune regulatory factor that enables tumor persistence under therapeutic pressure, supporting its development as a therapeutic target. Notably, small-molecule inhibitors and peptide disruptors targeting the MTDH-SND1 complex have shown preclinical efficacy and provide a promising framework for future therapeutic strategies in osteosarcoma[60, 61].

## Limitations

Notwithstanding the encouraging results, several limitations must be considered. First and foremost, the total number of samples included was limited, and the low incidence rate of osteosarcoma makes obtaining large-cohort data challenging. This constraint may limit the broader applicability of our findings, making larger-scale multi-center prospective studies essential for future confirmation and generalization of our results. Secondly, this study lacks direct genomic data support such as Circle-Seq or WGS. It is necessary to integrate more dimensions of clinical sample data in the future to deeply analyze the biological functions and clinical significance of ecDNA in osteosarcoma. Finally, although the comprehensive transcriptomic analysis results suggested a potential association between EGPSM and immune regulation, further experiments are needed to explore the underlying mechanisms.

## Conclusions

In conclusion, we investigated the relationship between ecDNA-related genes and osteosarcoma, observing that the presence of ecDNA disrupts the balance of T/NK cells and immune evasion molecules, thereby creating an “immune-cold” tumor microenvironment that promotes tumor progression. Furthermore, we constructed and validated a valuable prognostic model to effectively assess the tumor immune microenvironment, immunotherapy response, and prognosis of osteosarcoma patients. Notably, targeting MTDH effectively alleviated the tumor “immune-cold” microenvironment, inhibited osteosarcoma proliferation, invasion, and migration, and promoted apoptosis in osteosarcoma cells. This suggests that the EGPSM can serve as a potential therapeutic target and prognostic biomarker for osteosarcoma.

## Supplementary Information

Below is the link to the electronic supplementary material.

Figure S1. Functional enrichment analysis and immune infiltration analysis. **A** Differences in hallmark pathway activities between the high-risk and low-risk groups, according to the GSVA score. **B** The relative abundance of immune cells in the high-risk and low-risk groups. **C**-**D** Correlation between EGPSM-related genes and the abundance of immune cell infiltration. **E** The ESTIMATEScore, ImmuneScore, StromalScore, and TumorPurity were used to quantify the differences in immune status between the high-risk and low-risk groups. **F** Comparison of Statistically Significant Chemotherapy Drug IC50 Values Between the Two Risk Groups. ∗ *P* < 0.05, ∗∗ *P* < 0.01. (XLSX 16 KB)

Figure S2. The expression of immune factors in the high and low risk groups. **A** Multidimensional correlation plot illustrating the relationship between risk scores and the gene expression of immune checkpoints.**B** Heatmap illustrating the differences in gene expression of chemokines, interleukins, interferons, receptors, and other cytokines between the two risk groups. **C** Differences in MHC molecule expression between the high-risk and low-risk groups. ∗ *P*< 0.05, ∗∗ *P* < 0.01. (DOCX 16 KB)

Figure S3. Tumor mutations in high and low-risk groups. **A** The mutational landscape of patients in the high-risk group(left) and in the low-risk group(right). **B** The mutational landscape of patients in the high-risk group. (DOCX 15 KB)

Figure S4. Based on scRNA-seq analysis of the EGPSM. **A** nFeature_RNA, nCount_RNA, percent.mt, and percent HB in the samples before and after quality control filtering. **B** The proportion of different cells in each sample. **C** The heatmap shows the chromosomal mapping of CNVs in osteosarcoma cells inferred by InferCNV. **D**Cells were stratified into high and low EGPSM score groups using the median score as the cutoff. **E** The proportions of cell subpopulations were compared between the high and low ECScore groups. **F** Pseudotime plot showing the trajectory of all osteosarcoma cells. **G** Heatmap illustrating metabolic differences among the different EGPSM score groups. ∗ *P* < 0.05. (TIF 4911 KB)

Figure S5. ScRNA-seq analysis showed different characteristics of T cells related to the EGPSM score.**A**The proportion of different cells in each sample.**B** The proportions of T-cell subpopulations between the high- and low- risk score groups.**C** The interaction strength between osteosarcoma cells and T/NK cell subpopulations.**D** The bar chart displays the relative contribution of the high group (red) and the low group (cyan) to the information flow of each signaling pathway (ligand-receptor pair) in the CellChat analysis (TIF 3640 KB)

Figure S6. MTDH promotes tumor progression in osteosarcoma.**A** UMAP plot showing 9 gene expression density.**B**Overall survival differences between osteosarcoma patients with high and low MTDH expression in multiple datasets. **C** qRT-PCR was used to detect the mRNA expression of MTDH in osteosarcoma cell lines. *P < 0.05; ** *P* < 0.01; *** *P* < 0.001.(TIF 3297 KB)

Figure S7. MTDH promotes tumor progression in osteosarcoma. **A**-**B** The efficiency of stable MTDH overexpression in 143B and U2OS cells was validated by qRT-PCR and Western blot assays, respectively. **C** CCK-8 assay tested the effect of MTDH overexpression on the proliferation ability of 143B and U2OS cells. **D** Colony formation assay and quantitative analysis were performed to evaluate the impact of increased MTDH expression on the tumorigenic potential of osteosarcoma cells. **E** Flow cytometry quantified the percentage of apoptotic cells, showing that MTDH overexpression reduced cell apoptosis compared to the control group. **F**-**G** Wound healing assay was conducted to evaluate the effect of MTDH overexpression on the migration ability of 143B and U2OS cells. **H** The impact of MTDH overexpression on cell migration and invasion was further investigated using Transwell assays in 143B and U2OS cells. * *P* < 0.05; ** *P*< 0.01; *** *P* < 0.001. (TIF 5242 KB)

Figure S8. MTDH disruption promotes CD8+ T cell-mediated antitumor immunity suppressing osteosarcoma cells growth. **A** A T cell-mediated tumor cell killing assay was performed by co-culturing activated CD8⁺ T cells with 143B or U2OS cells transfected with either a control plasmid or an MTDH-overexpression plasmid for 3 days. The left panel shows representative images of surviving tumor cells stained with crystal violet, and the right panel presents the quantification of the staining intensity of the surviving cells. **B** CD8⁺ T cells were co-cultured for 48 h with 143B and U2OS cells transfected with either a control plasmid or an MTDH-overexpression plasmid. The fractions of GzmB⁺, IFN-γ⁺, and Perforin⁺ cells within the CD8⁺ T cell population were then quantified by flow cytometry.**C** Box plots showing the percentage of total CD8⁺ T cells, as well as the fractions of GzmB⁺, IFN-γ⁺, and Perforin⁺ CD8⁺ T cells, among different groups based on flow cytometry analysis of the orthotopic mouse tibial model.* *P* < 0.05; ** *P*  < 0.01; *** *P*  < 0.001. (TIF 1788 KB)

Figure S9. MTDH disruption promotes CD8+ T-cell-mediated antitumor immunity suppressing OS cells growth. **A** The bar chart displays the magnitude of change for genes significantly affected after virtual MTDH knockout. **B**-**C** (B-C) Cellular Component (CC) enrichment analysis **B** and Molecular Function (MF) enrichment analysis **C** after MTDH virtual knockout.**D** Immune infiltration analysis in the GSE21257 dataset revealed a correlation between MTDH and the infiltration level of CD8+ T cells.(TIF 1772 KB)

Table S1. ecDNA-related genes. (TIF 8378 KB)

Table S2. List of primers and siRNA sequences used in this article. (TIF 3242 KB)

Table S3. List of antibodies used in this article.(TIF 1646 KB)

## Data Availability

The data generated and analyzed in the current study are available from the corresponding author upon reasonable request.
