# Supplementary material for: Integrating single-cell and bulk transcriptomes to reveal prognostic and immunological features of ecDNA-related genes in osteosarcoma
Source: Cancer Immunol Immunother. 2026 Apr 28;75(5):159. doi: 10.1007/s00262-026-04383-2 (PMC13125642; doi:10.1007/s00262-026-04383-2)
Supplement: Supplementary file 2 — Figure S2. The expression of immune factors in the high and low risk groups. A Multidimensional correlation plot illustrating the relationship between risk scores and the gene expression of immune checkpoints.B Heatmap illustrating the differences in gene expression of chemokines, interleukins, interferons, receptors, and other cytokines between the two risk groups. C Differences in MHC molecule expression between the high-risk and low-risk groups. ∗ P< 0.05, ∗∗ P < 0.01. (DOCX 16 KB) [file 262_2026_4383_MOESM2_ESM.docx]

**Supplementary information**

**Supplementary Table S2** List of primers and siRNA sequences used in this article.

| **Plasmid** | | |
| --- | --- | --- |
| **Gene** | **Primer** | **Sequence (5′-3′)** |
| MTDH | Forward | ATGGCTGCACGGAGCTGGCA |
|  | Reverse | ATCACGTTTCTCGTCTGGCTTTTTTCTTC |
| MYC | Forward | ATGCCCCTCAACGTTAGCTTCACC |
|  | Reverse | TTACGCACAAGAGTTCCGTAGCTGTTC |
| **qPCR** | | |
| **Gene** | **Primer** | **Sequence (5′-3′)** |
| MTDH | Forward | TCTGCTGATCCCAACTCTG |
|  | Reverse | GCTCCCTCTCCCTTTTCTT |
| GAPDH | Forward | AACGGATTTGGTCGTATTG |
|  | Reverse | GGAAGATGGTGATGGGATT |
| MYC | Forward | GGCTCCTGGCAAAAGGTCA |
|  | Reverse | CTGCGTAGTTGTGCTGATGT |
| **siRNA** | | |
| MTDH | Forward | CGTGATAAGGTGCTGACTGAT |
