# Supplementary material for: Integrating single-cell and bulk transcriptomes to reveal prognostic and immunological features of ecDNA-related genes in osteosarcoma
Source: Cancer Immunol Immunother. 2026 Apr 28;75(5):159. doi: 10.1007/s00262-026-04383-2 (PMC13125642; doi:10.1007/s00262-026-04383-2)
Supplement: Supplementary file 3 — Figure S3. Tumor mutations in high and low-risk groups. A The mutational landscape of patients in the high-risk group(left) and in the low-risk group(right). B The mutational landscape of patients in the high-risk group. (DOCX 15 KB) [file 262_2026_4383_MOESM3_ESM.docx]

**Supplementary information**

**Supplementary Table S3.** List of antibodies used in this article.

| **Antibody** | **Catalog Number** | **Source** |
| --- | --- | --- |
| CD8 | 344721 | BioLegend |
| GZMB | 396413 | BioLegend |
| IFN-γ | 502527 | BioLegend |
| perforin | 308105 | BioLegend |
| FIlC anti-mouse CD3 | 100203 | BioLegend |
| APCanti-mouse CD8a | 100711 | BioLegend |
| Brilliant Violet 421rM anti-human/mouse Granzyme B Recombinant | 396413 | BioLegend |
| PE anti-mouse Perforin | 154305 | BioLegend |
| PE/Cyanine7 anti-mouse IFN-γ | 505825 | BioLegend |
| Alexa Fluor® 700 anti-mouse CD45 | 103127 | BioLegend |
