# Supplementary figures and images for: Integrating single-cell and bulk transcriptomes to reveal prognostic and immunological features of ecDNA-related genes in osteosarcoma
Source: Cancer Immunol Immunother. 2026 Apr 28;75(5):159. doi: 10.1007/s00262-026-04383-2 (PMC13125642; doi:10.1007/s00262-026-04383-2)

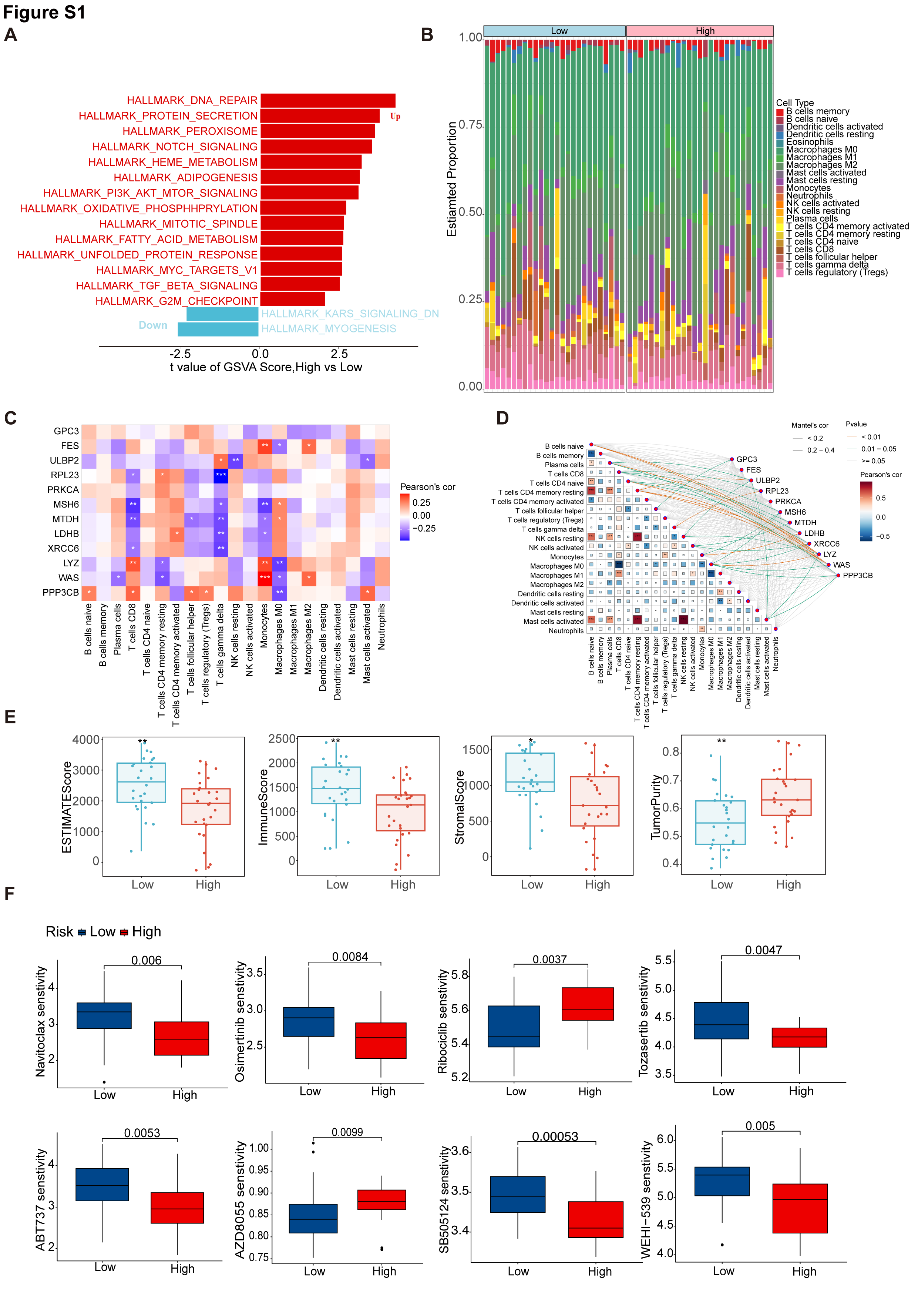

Supplement: Supplementary file 4 — Figure S4. Based on scRNA-seq analysis of the EGPSM. A nFeature_RNA, nCount_RNA, percent.mt, and percent HB in the samples before and after quality control filtering. B The proportion of different cells in each sample. C The heatmap shows the chromosomal mapping of CNVs in osteosarcoma cells inferred by InferCNV. DCells were stratified into high and low EGPSM score groups using the median score as the cutoff. E The proportions of cell subpopulations were compared between the high and low ECScore groups. F Pseudotime plot showing the trajectory of all osteosarcoma cells. G Heatmap illustrating metabolic differences among the different EGPSM score groups. ∗ P < 0.05. (TIF 4911 KB) [file 262_2026_4383_MOESM4_ESM.tif]

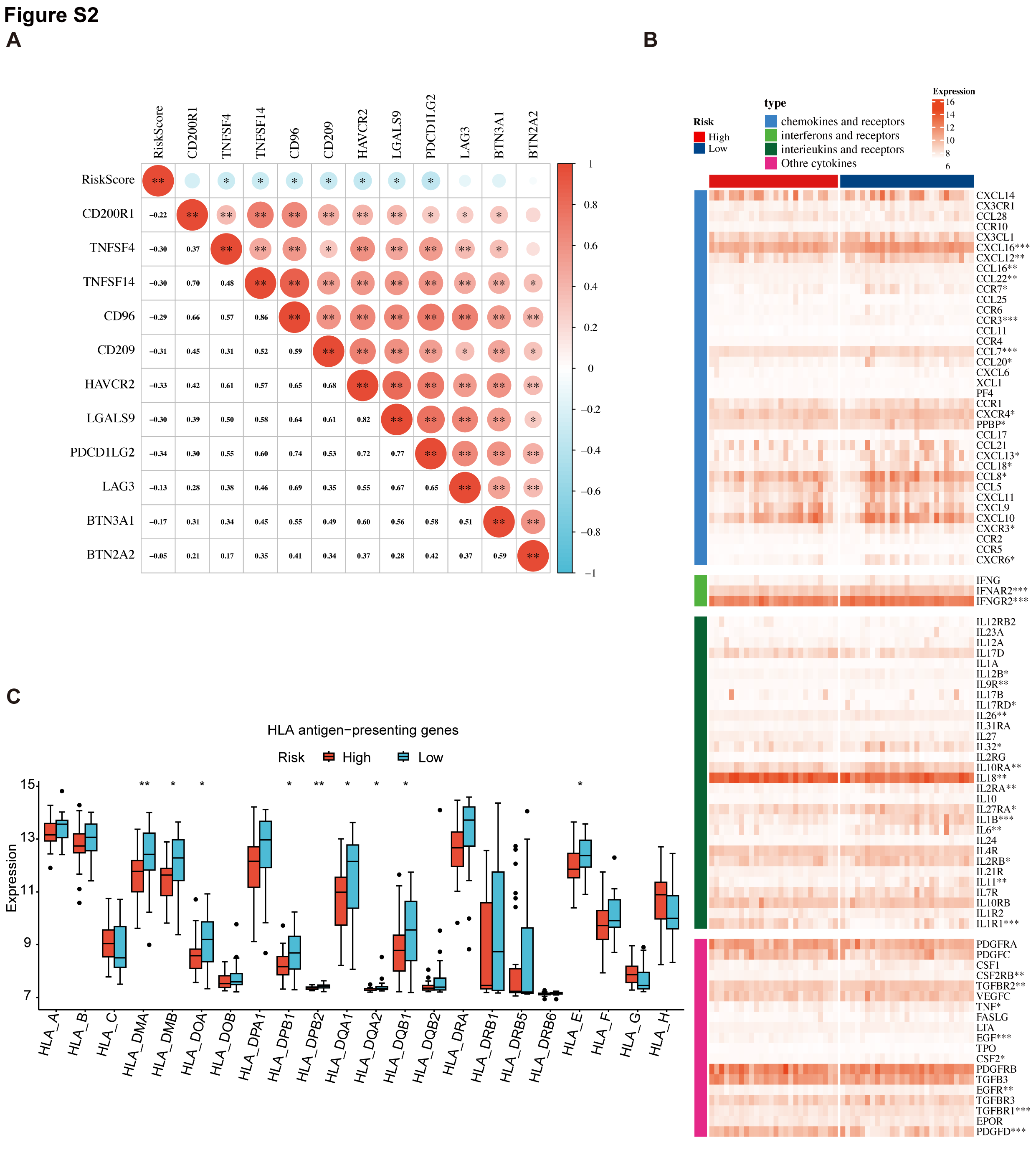

Supplement: Supplementary file 5 — Figure S5. ScRNA-seq analysis showed different characteristics of T cells related to the EGPSM score.AThe proportion of different cells in each sample.B The proportions of T-cell subpopulations between the high- and low- risk score groups.C The interaction strength between osteosarcoma cells and T/NK cell subpopulations.D The bar chart displays the relative contribution of the high group (red) and the low group (cyan) to the information flow of each signaling pathway (ligand-receptor pair) in the CellChat analysis (TIF 3640 KB) [file 262_2026_4383_MOESM5_ESM.tif]

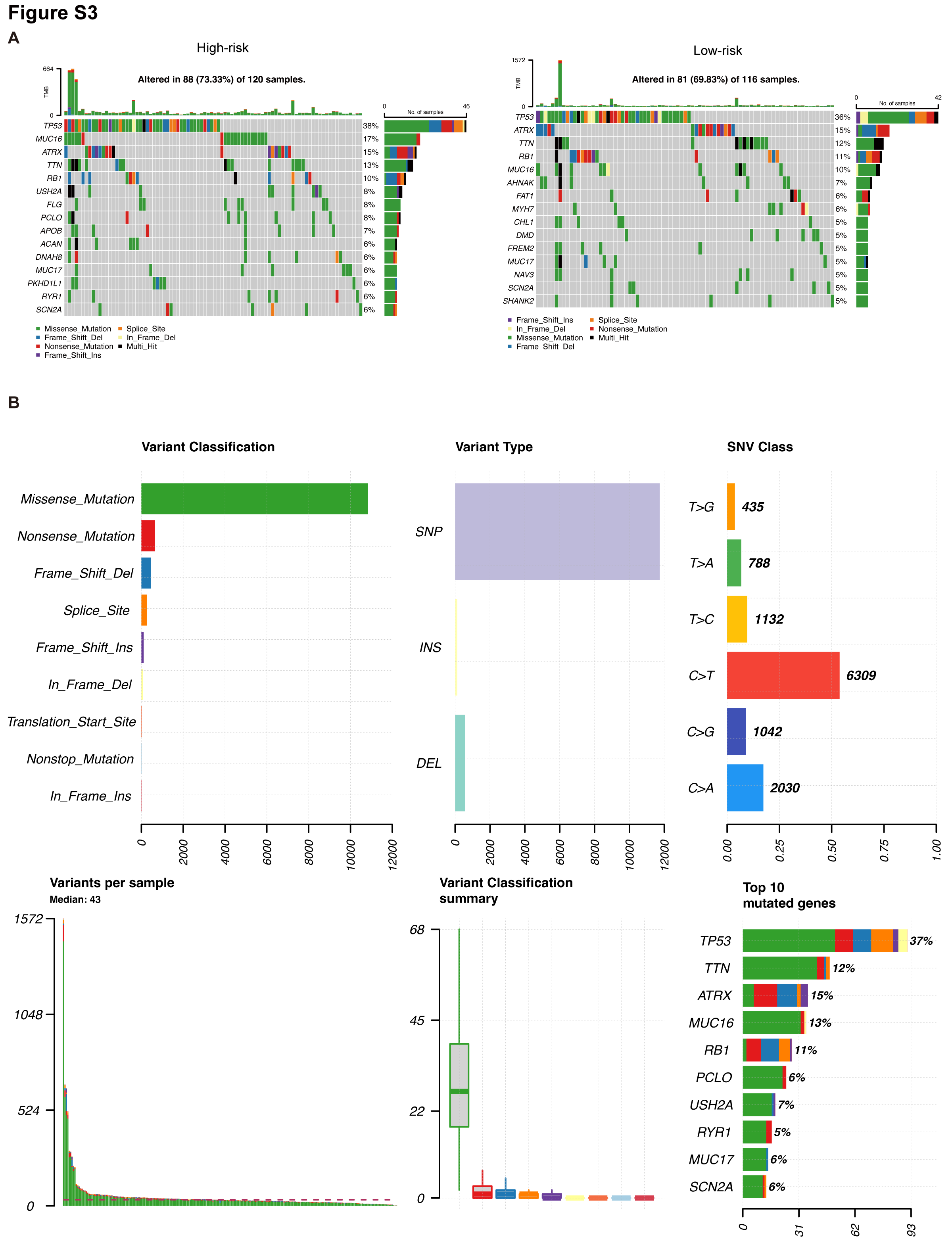

Supplement: Supplementary file 6 — Figure S6. MTDH promotes tumor progression in osteosarcoma.A UMAP plot showing 9 gene expression density.BOverall survival differences between osteosarcoma patients with high and low MTDH expression in multiple datasets. C qRT-PCR was used to detect the mRNA expression of MTDH in osteosarcoma cell lines. *P < 0.05; ** P < 0.01; *** P < 0.001.(TIF 3297 KB) [file 262_2026_4383_MOESM6_ESM.tif]

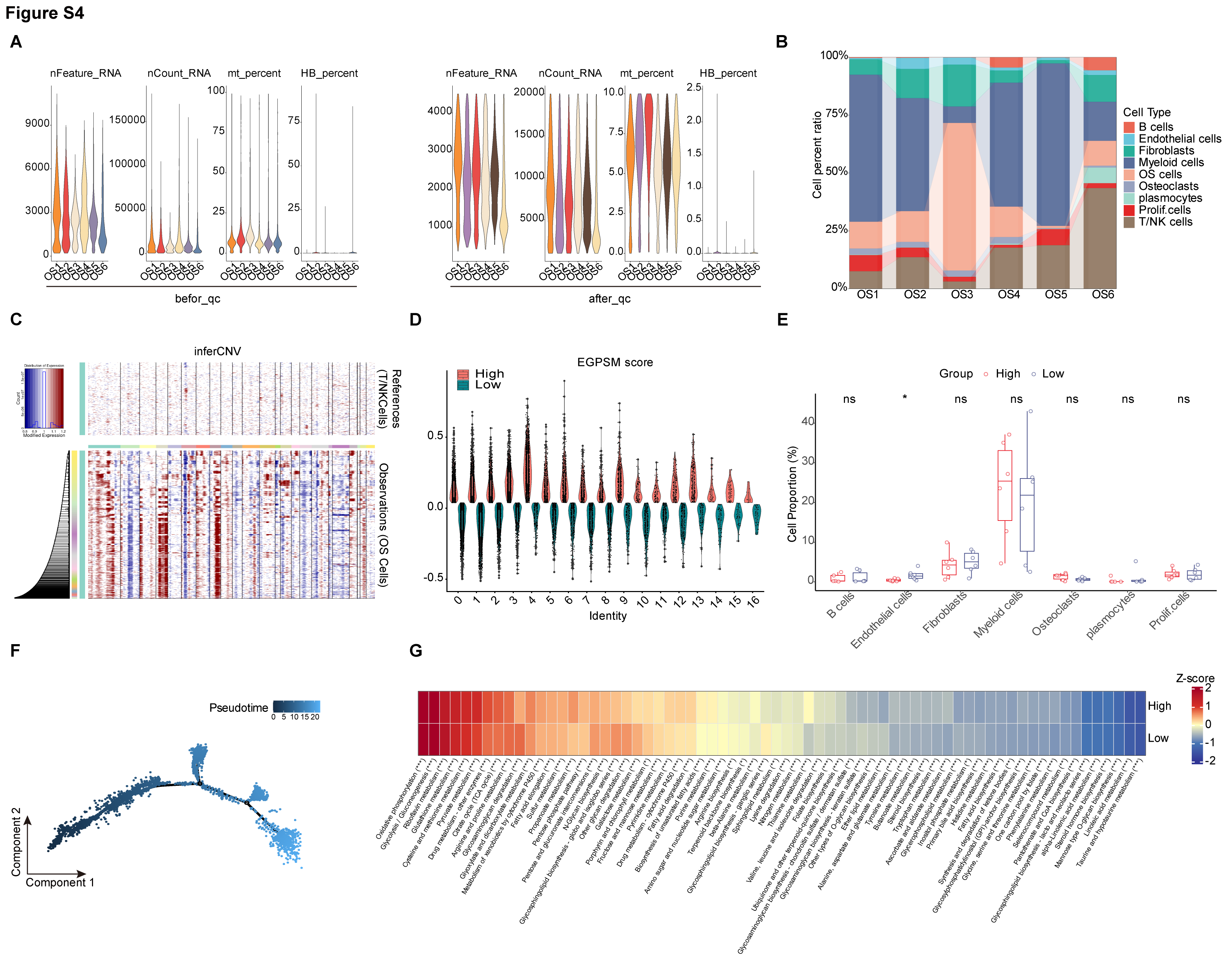

Supplement: Supplementary file 7 — Figure S7. MTDH promotes tumor progression in osteosarcoma. A-B The efficiency of stable MTDH overexpression in 143B and U2OS cells was validated by qRT-PCR and Western blot assays, respectively. C CCK-8 assay tested the effect of MTDH overexpression on the proliferation ability of 143B and U2OS cells. D Colony formation assay and quantitative analysis were performed to evaluate the impact of increased MTDH expression on the tumorigenic potential of osteosarcoma cells. E Flow cytometry quantified the percentage of apoptotic cells, showing that MTDH overexpression reduced cell apoptosis compared to the control group. F-G Wound healing assay was conducted to evaluate the effect of MTDH overexpression on the migration ability of 143B and U2OS cells. H The impact of MTDH overexpression on cell migration and invasion was further investigated using Transwell assays in 143B and U2OS cells. * P < 0.05; ** P< 0.01; *** P < 0.001. (TIF 5242 KB) [file 262_2026_4383_MOESM7_ESM.tif]

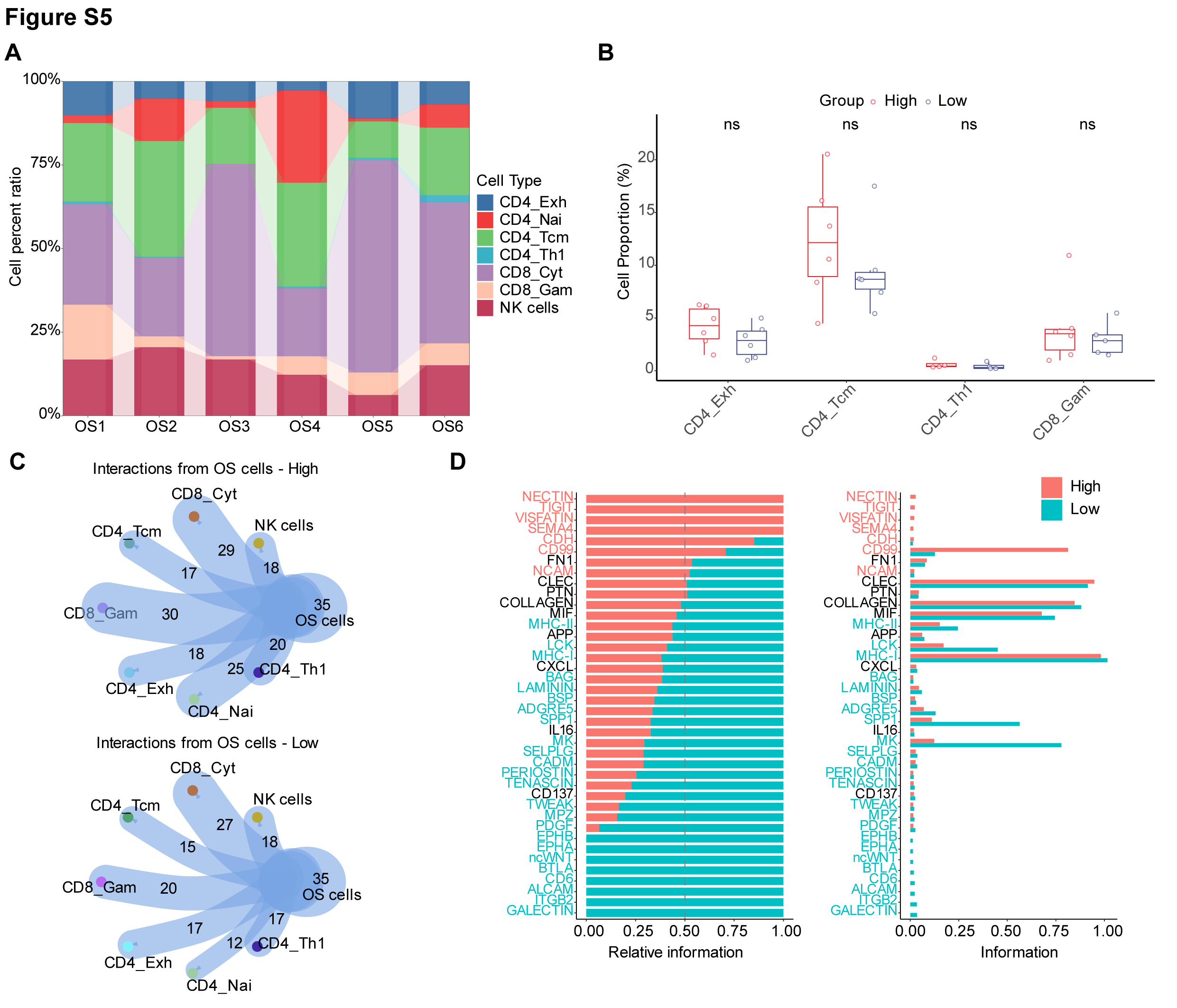

Supplement: Supplementary file 8 — Figure S8. MTDH disruption promotes CD8+ T cell-mediated antitumor immunity suppressing osteosarcoma cells growth. A A T cell-mediated tumor cell killing assay was performed by co-culturing activated CD8⁺ T cells with 143B or U2OS cells transfected with either a control plasmid or an MTDH-overexpression plasmid for 3 days. The left panel shows representative images of surviving tumor cells stained with crystal violet, and the right panel presents the quantification of the staining intensity of the surviving cells. B CD8⁺ T cells were co-cultured for 48 h with 143B and U2OS cells transfected with either a control plasmid or an MTDH-overexpression plasmid. The fractions of GzmB⁺, IFN-γ⁺, and Perforin⁺ cells within the CD8⁺ T cell population were then quantified by flow cytometry.C Box plots showing the percentage of total CD8⁺ T cells, as well as the fractions of GzmB⁺, IFN-γ⁺, and Perforin⁺ CD8⁺ T cells, among different groups based on flow cytometry analysis of the orthotopic mouse tibial model.* P < 0.05; ** P < 0.01; *** P < 0.001. (TIF 1788 KB) [file 262_2026_4383_MOESM8_ESM.tif]

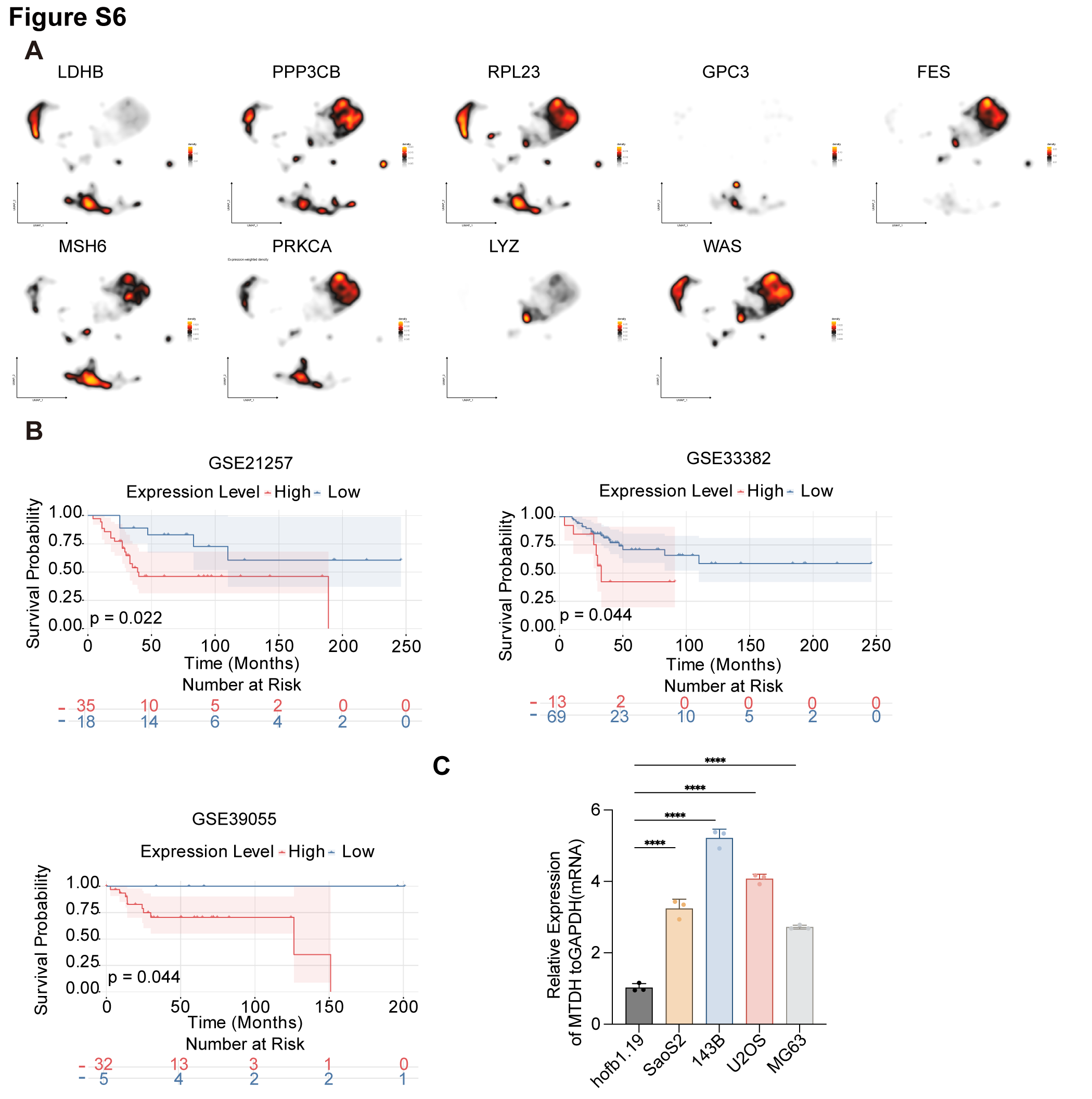

Supplement: Supplementary file 9 — Figure S9. MTDH disruption promotes CD8+ T-cell-mediated antitumor immunity suppressing OS cells growth. A The bar chart displays the magnitude of change for genes significantly affected after virtual MTDH knockout. B-C (B-C) Cellular Component (CC) enrichment analysis B and Molecular Function (MF) enrichment analysis C after MTDH virtual knockout.D Immune infiltration analysis in the GSE21257 dataset revealed a correlation between MTDH and the infiltration level of CD8+ T cells.(TIF 1772 KB) [file 262_2026_4383_MOESM9_ESM.tif]

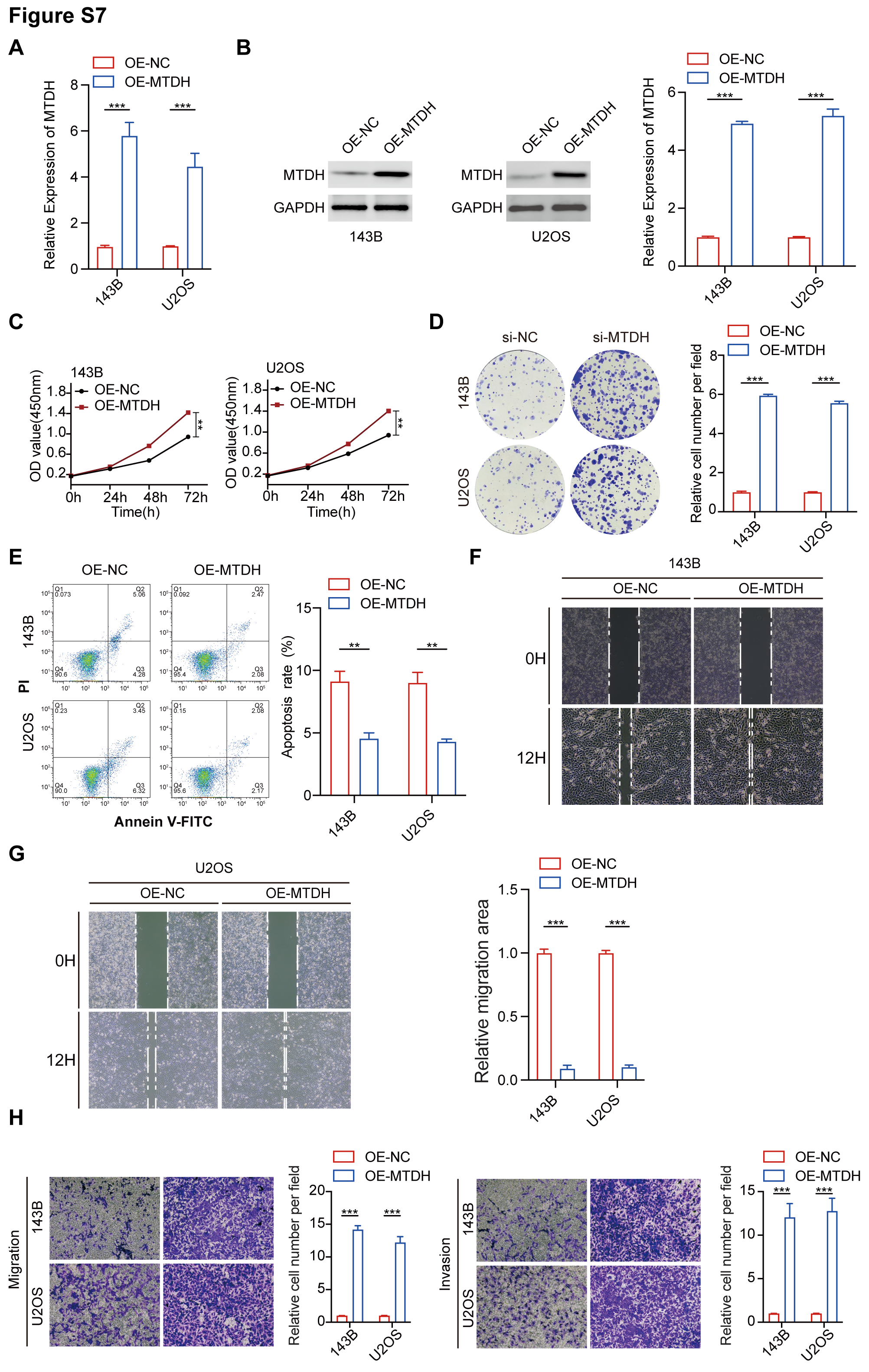

Supplement: Supplementary file 10 — Table S1. ecDNA-related genes. (TIF 8378 KB) [file 262_2026_4383_MOESM10_ESM.tif]

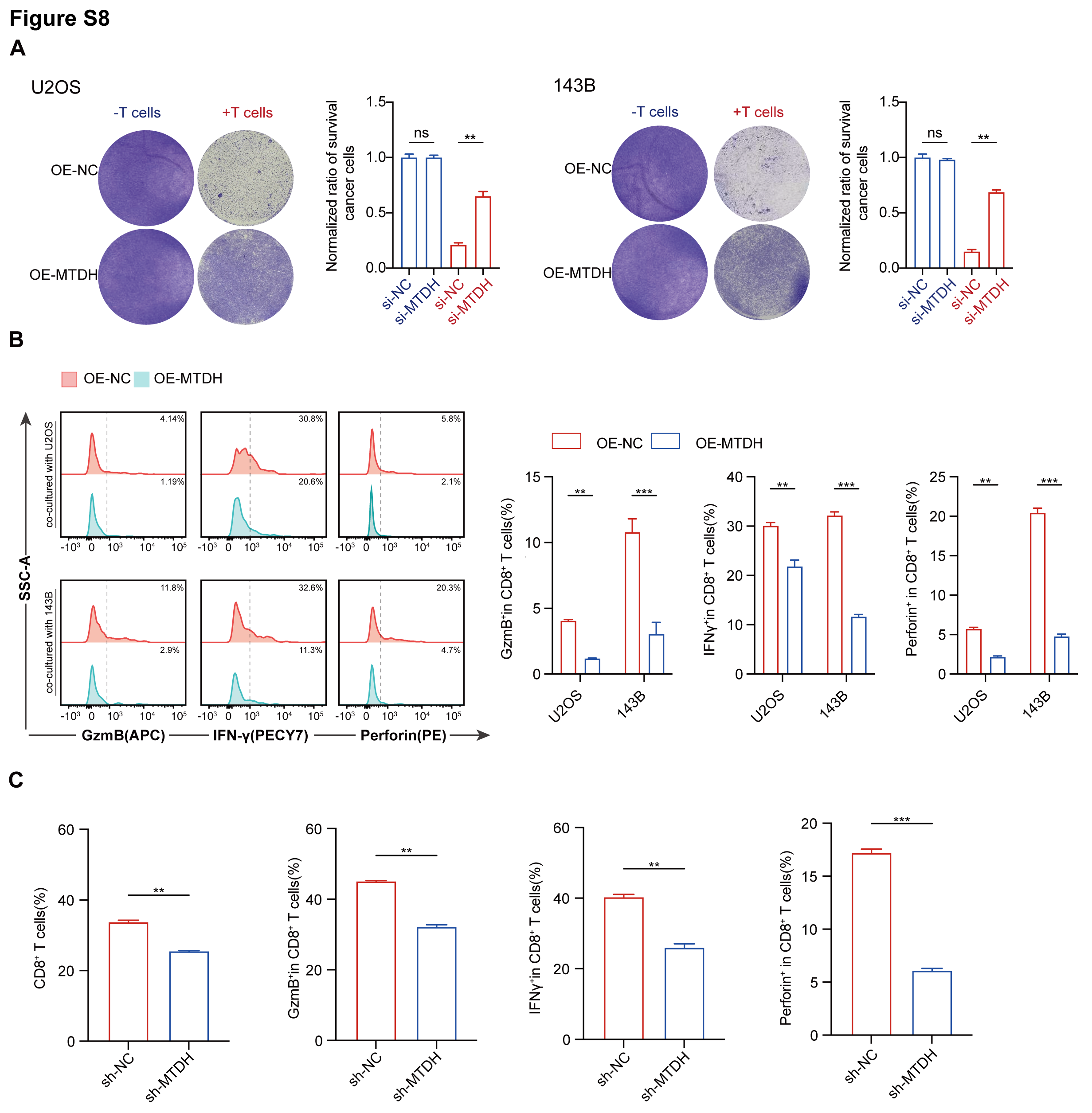

Supplement: Supplementary file 11 — Table S2. List of primers and siRNA sequences used in this article. (TIF 3242 KB) [file 262_2026_4383_MOESM11_ESM.tif]

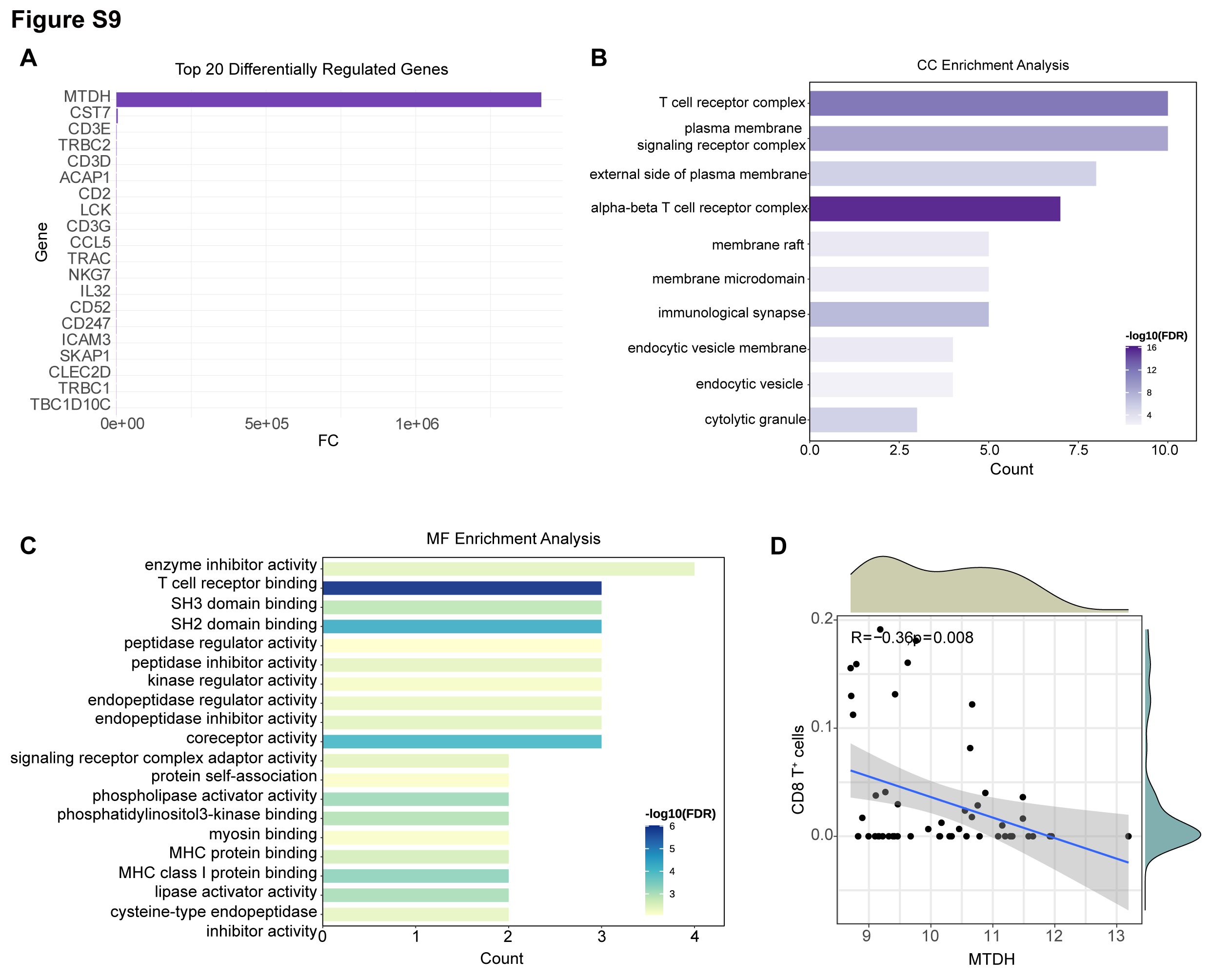

Supplement: Supplementary file 12 — Table S3. List of antibodies used in this article.(TIF 1646 KB) [file 262_2026_4383_MOESM12_ESM.tif]
